# Hot spot profiles of SARS-CoV-2 and human ACE2 receptor protein protein interaction obtained by density functional tight binding fragment molecular orbital method

**DOI:** 10.1038/s41598-020-73820-8

**Published:** 2020-10-08

**Authors:** Hocheol Lim, Ayoung Baek, Jongwan Kim, Min Sung Kim, Jiaxin Liu, Ky-Youb Nam, JeongHyeok Yoon, Kyoung Tai No

**Affiliations:** 1 Department of Biotechnology, Yonsei University, Seoul, Republic of Korea; 2 The Interdisciplinary Graduate Program in Integrative Biotechnology and Translational Medicine, Yonsei University, Incheon, Republic of Korea; 3 Bioinformatics and Molecular Design Research Center (BMDRC), Incheon, Republic of Korea; 4 Pharos I&BT Co., Ltd., Anyang-si, Gyeonggi-do Republic of Korea

**Keywords:** Computational biology and bioinformatics, Protein analysis, Viral proteins

## Abstract

The prevalence of a novel β-coronavirus (SARS-CoV-2) was declared as a public health emergency of international concern on 30 January 2020 and a global pandemic on 11 March 2020 by WHO. The spike glycoprotein of SARS-CoV-2 is regarded as a key target for the development of vaccines and therapeutic antibodies. In order to develop anti-viral therapeutics for SARS-CoV-2, it is crucial to find amino acid pairs that strongly attract each other at the interface of the spike glycoprotein and the human angiotensin-converting enzyme 2 (hACE2) complex. In order to find hot spot residues, the strongly attracting amino acid pairs at the protein–protein interaction (PPI) interface, we introduce a reliable inter-residue interaction energy calculation method, FMO-DFTB3/D/PCM/3D-SPIEs. In addition to the SARS-CoV-2 spike glycoprotein/hACE2 complex, the hot spot residues of SARS-CoV-1 spike glycoprotein/hACE2 complex, SARS-CoV-1 spike glycoprotein/antibody complex, and HCoV-NL63 spike glycoprotein/hACE2 complex were obtained using the same FMO method. Following this, a 3D-SPIEs-based interaction map was constructed with hot spot residues for the hACE2/SARS-CoV-1 spike glycoprotein, hACE2/HCoV-NL63 spike glycoprotein, and hACE2/SARS-CoV-2 spike glycoprotein complexes. Finally, the three 3D-SPIEs-based interaction maps were combined and analyzed to find the consensus hot spots among the three complexes. As a result of the analysis, two hot spots were identified between hACE2 and the three spike proteins. In particular, E37, K353, G354, and D355 of the hACE2 receptor strongly interact with the spike proteins of coronaviruses. The 3D-SPIEs-based map would provide valuable information to develop anti-viral therapeutics that inhibit PPIs between the spike protein of SARS-CoV-2 and hACE2.

## Introduction

The novel coronavirus SARS-CoV-2 (2019-nCoV) was first identified in Wuhan in China’s Hubei province, and has been categorized as a human pathogen since December 2019^1–3^. It causes coronavirus disease 2019 (COVID-19), characterized by fever, shortness of breath, severe respiratory illness, and pneumonia. The SARS-CoV-2 is a β-coronavirus, which is one of four genera (α, β, γ, δ) of coronaviruses (CoVs). α- and β-CoVs can infect mammals, whereas γ- and δ-CoVs tend to infect birds^4^. Previously, two α-coronaviruses (HCoV-229E and HCoV-NL63) and four β-coronaviruses (HCoV-HKU1, HCoV-OC43, SARS-CoV, and MERS-CoV) had been identified as human viruses^4^.

SARS-CoV-2 makes use of a densely glycosylated spike (S) protein to invade host cells. The S protein is a trimeric class I fusion protein and undergoes a structural rearrangement to fuse the viral membrane with the host cell membrane^3,5,6^. The S1 subunit of the S protein binds to a host cell receptor and the receptor-binding domain (RBD) of S1 undergoes hinge-like conformational changes that transiently conceal or reveal the determinants of receptor binding^3^. SARS-CoV-2 could possibly use angiotensin-converting enzyme 2 (hACE2), the same receptor as SARS-CoV^4^ and HCoV-NL63. Since the essential function of the S protein is to penetrate host cells, it is considered as the optimal target for the prevention of cell infection. For this reason, S protein-targeted antibody-mediated neutralization has been considered as a suitable treatment for SARS-CoV diseases. Therefore, the hot spot analysis on the interface between the RBD domain of the S1 subunit and the hACE2 receptor would provide crucial information for antibody engineering and for small-molecular drug development.

To investigate protein–protein interactions (PPIs) between hACE2 and the RBD domain of S1 subunit at the molecular level, an ab initio quantum mechanical (QM) method was introduced. This method was used to obtain the most accurate information on the PPIs through analysis of the wave function obtained from the QM calculation, especially of the fragment molecular orbital (FMO) approximation method. Even with the FMO method, the calculations in a biomolecular system need a huge amount of computer resources. In order to obtain results within a reasonable computation time while maintaining a certain degree of accuracy of ab initio MO, we introduced the density functional tight-binding (DFTB) method, which is an efficient parameterized QM method and is expected to exhibit reasonable accuracy at a remarkably reduced computational cost^7^. The FMO method is one of various linear-scaling methods to reduce the huge computational cost of QM calculations by the fragmentation of target molecules. The energies of fragment and their pairs are computed in the embedding electrostatic potential^8^. Recently, the FMO method has been combined with DFTB, and the polarizable continuum model (PCM) was introduced to consider the effect of a solvent on a model system^9^. Pair interaction energies (PIEs) among the fragments of the model system from the FMO-DFTB/PCM method correlate well with PIEs from ab initio DFT FMO/PCM and with an ignition Møller-Plesset perturbation theory (MP2) FMO/PCM^9^. In our earlier work, we investigated PPIs between programmed cell death 1 and its ligand PD-L1 using FMO-MP2/PCM and the results efficiently explained the experimental site-directed mutagenesis data^10^.

In this work, to find common hot spot amino acids on the interfaces between the RBD domain and hACE2 of the three complexes, RBD-SARS-CoV-2/hACE2 (twelve experimental structural data), RBD-SARS-CoV-1/hACE2 (four experimental structural data), and RBD-HCoV-NL63/hACE2 (one experimental structural data), we performed FMO-DFTB3/D/PCM calculations. To visualize the interaction energy and the distance of the interacting amino acid pairs, the FMO/3D-SPIEs analysis tool was introduced. To narrow down the hot spot region, we also performed the same calculation with RBD-SARS-CoV-1/antibody complexes (five experimental structural data). Based on the FMO/3D-SPIEs results, we constructed 3D-SPIEs-based interaction maps of the hACE2 and RBD domains from SARS-CoV-1, HCoV-NL63, and SARS-CoV-2.

In order to validate the PPI predictability of the FMO-DFTB3/D/PCM results, we compared them with the site-directed mutagenesis results. Consequently, we summarized the FMO-DFTB3/D/PCM/3D-SPIEs results as interaction maps and found the hot spot regions in RBD-SARS-CoV-2 and hACE2 at a QM level.

## Computational methods

All experimental structures calculated in this work are summarized in Table 1. All missing side chains were filled using Prime implemented in Maestro program^11^. Hydrogen atoms were added to the crystal structures at pH 7.0 and their positions were optimized with the PROPKA function implemented in Maestro program^12^. Water molecules in the crystal structures were included in the FMO calculations to explore their roles in PPIs. In the FMO calculations, all N-acetylglucosamine (GlcNAc) residues in the RBD domains and hACE2 were included, only RBD domains of three coronaviruses were included, and only Fv domains of antibodies that bind to RBD domains were included.

**Table 1:** Summary of 23 experimental structures analyzed in this work.

Class	Complex	PDB ID	Total atom number	Calculation time (s)	Methods	Resolution (Å)
SARS-CoV-1	hACE2/SARS-CoV-1	2AJF	12,458	4739	X-ray	2.90
	hACE2/SARS-CoV-1	3D0G	12,299	4369	X-ray	2.80
	hACE2/SARS-CoV-1	3D0H	12,304	4536	X-ray	3.10
	hACE2/SARS-CoV-1	3D0I	12,301	4432	X-ray	2.90
	hACE2/SARS-CoV-1	3SCI	12,256	4521	X-ray	2.90
	hACE2/SARS-CoV-1	3SCJ	12,257	4403	X-ray	3.00
	hACE2/SARS-CoV-1	3SCK	12,234	4444	X-ray	3.00
	hACE2/SARS-CoV-1	3SCL	12,236	4219	X-ray	3.00
	hACE2/SARS-CoV-1	6ACG	12,474	4634	Cryo-EM	5.40
	hACE2/SARS-CoV-1	6ACJ	12,474	4759	Cryo-EM	4.20
	hACE2/SARS-CoV-1	6ACK	12,473	4632	Cryo-EM	4.50
	hACE2/SARS-CoV-1	6CS2	11,566	6697	Cryo-EM	4.40
SARS-CoV-1 antibody	SARS-CoV-1/80R	2GHW	6534	1228	X-ray	2.30
	SARS-CoV-1/m395	2DD8	6318	1802	X-ray	2.30
	SARS-CoV-1/S230 state 1	6NB6	16,284	6927	Cryo-EM	4.20
	SARS-CoV-1/S230 state 2	6NB7	6717	2276	Cryo-EM	4.50
	SARS-CoV-1/F26G19	3BGF	9284	2871	X-ray	3.00
HCoV-NL63	hACE2/HCoV-NL63	3KBH	11,243	3587	X-ray	3.31
SARS-CoV-2	hACE2/SARS-CoV-2	6M17	12,681	8551	X-ray	2.90
	hACE2/SARS-CoV-2	6VW1	12,896	8477	X-ray	2.68
	hACE2/SARS-CoV-2	6LGZ	13,593	13,018	X-ray	2.50
	hACE2/SARS-CoV-2	6M0J	12,863	8707	X-ray	2.45
SARS-CoV-2 antibody	SARS-CoV-2/B38	7BZ5	7611	4082	X-ray	1.84

All FMO calculations were performed with the version Feb 14, 2018 GAMESS^13^. The two-body FMO method was applied to all calculations in this work for the FMO2/DFTB3 method; this is a recent extension of the self-consistent-charge density functional tight-binding method and derived via a third-order expansion of the DFT method^9^. DFTB3 calculations were performed using the 3OB parameter set^14,15^, and the UFF-type dispersion correction (DFTB3/D)^16,17^. Due to the exchange-repulsion term is not computed in DFTB, the EX terms are all zero (see Supplementary Table S2-S25). The polarizable continuum model (PCM), an implicit solvation model, was employed with the explicitly expressed water molecules present in the X-ray crystal and cryo-EM structures. All input files were prepared in compliance with the hybrid orbital projection (HOP) scheme fragmentation^18^. Each residue and water molecule was defined as one fragment. Two cysteine residues forming the disulfide bridge were defined as one fragment, and GlcNAc, with which the asparagine residue formed covalent bonds, was defined as one fragment. All 3D-SPIEs results were generated with the reported protocol^10^. In the protocol, we selected only PIEs within a specific distance (5.0 Å) between two fragments, which reflected the distance used for the approximate of electrostatic potential in FMO method^19^. We considered the interaction with an PIE more stable than − 3.0 kcal/mol to be significant on the basis of previous reports^10,20,21^.

## Results and discussion

To investigate PPIs between hACE2 and RBD domains of the three coronaviruses, we collected 23 experimental structures summarized in Table 1 and performed FMO-DFTB3/D/PCM calculations for all the experimental structures. Due to structural arrangements from mutations summarized in Table S1, we collected all available structures to consider them together. Subsequently, we performed hot spot analysis using the FMO/3D-SPIEs tool.

### Hot spot region between hACE2 and RBD-SARS-CoV-1

**Figure 1:**
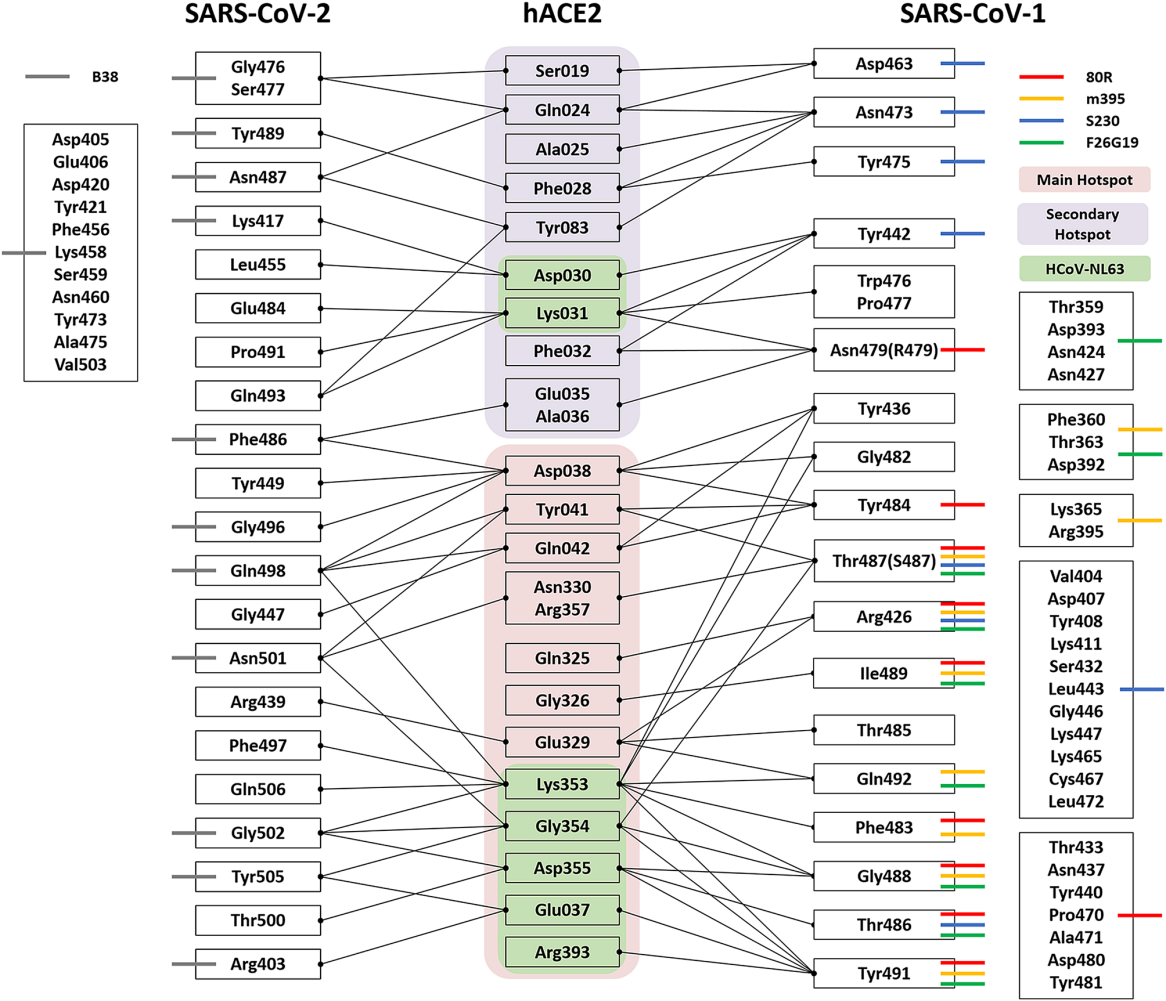
Interaction map among hACE2, SAS-CoV-1, HCoV-NL63, SARS-CoV-2, and SARS-CoV-1 antibodies. 3D-SPIEs-based interaction map consists of hACE2, RBD domains from SARS-CoV-1, HCoV-NL63, SARS-CoV-2, four SARS-CoV-1 antibodies, and one SARS-CoV-2 antibody. The interactions between hACE2 and RBD domain from SARS-CoV-2 are shown in the left-hand with black arrows. The interactions between one antibody (B38) and RBD domain from SARS-CoV-2 are shown in the left-hand with color bars. The interactions in hACE2 and RBD domain from SARS-CoV-1 are shown in the right-hand with black arrows. The interactions between hACE2 and HCoV-NL63 are colored in green in the middle. The interactions between four antibodies (80R, m395, S320, and F26G13) and RBD domain from SAR-CoV-1 are shown in the right-hand with color bars. the main hot spot region is colored in light red, and the secondary hot spot region in hACE2 is colored in light blue, and All interactions shown in this map have attractive PIE value more stable than − 3.0 kcal/mol, whose magnitudes are ignored.

In order to investigate the hot spot region in the RBD of the SAR-CoV-1 and hACE2 receptor complex, we performed FMO calculations on 12 RBD-SARS-CoV-1/hACE2 complexes (Supplementary Table S2-S13). We summarized the FMO results in Fig. 1. In 12 RBD-SARS-CoV-1/hACE2 complexes, the FMO results detected 69 amino acid pairs as the union of the amino acid pair members from all the 12 complex structures. Among the 69 amino acid pairs, all 12 complexes have two common amino acid pairs, (1) a cation–π interaction between K353 in hACE2 and F483 in RBD-SARS-CoV-1, and (2) a hydrogen bond interaction between D355 in hACE2 and T486 in RBD-SARS-CoV-1. Ten of the twelve complexes have a common amino acid pair, an electrostatic interaction between the carboxyl oxygen of K353 and T487 and Y491 in RBD-SARS-CoV-1. Another 10 complexes also have an electrostatic interaction between R357 in hACE2 and T487 in RBD-SARS-CoV-1. Nine of the twelve complexes have six common amino acid pairs in hACE2/RBD: S19/D463, F28/Y475, Q325/R426, E329/R426, K353/Q492, and D355/G488. Eight of the twelve complexes have three common amino acid pairs: Q42/Y484, Y83/N473, and G354/G488. Seven of the twelve complexes have one common amino acid pair: D38/Y436. Six of the twelve complexes have one common amino acid pair: D38/G482.

The amino acid pairs that contributed to the stability of the complexes are well correlated with the published site-directed mutagenesis studies. Wu et al. reported that 2 mutations (D38A and Y41F) in hACE2 and 3 mutations (Y491A, T487A, and T487S) in RBD-SARS-CoV-1 lowered the binding affinity^22^. Li et al*.* reported that 10 mutations in hACE2 affected the inhibition of interactions with the SARS-CoV-1 spike protein (Q24K/K26E, K31D, Y41A, K68D, K353D, K353A, K353D, D355A, R357A, and R393A)^23^. When comparing the 69 amino acid pairs of this study with the mutagenesis experimental results from two papers, it was confirmed that 32 of the 69 amino acid pairs correlated with the experimental results.

The changes in the binding affinity between the proteins that form a complex by mutation can be explained by comparing the structural changes (i.e. changes in the amino acid pairs that contribute to the increase or decrease of the binding affinity) of the mutated proteins with those of the wild-type proteins. Qu et al*.* reported that the N479K/T487S mutation on RBD-SARS-CoV-1 lowers the binding affinity^24^. One complex (PDB ID: 3D0H) has the T487S mutation in RBD-SARS-CoV-1. T487 in WT RBD-SARS-CoV-1 attractively interacts with 6 amino acids, Y41, G326, N330, G354, F356, and R357, whereas S487 in the mutated complex attractively interacts with only 3 amino acids, N330, G354, and R357. Wu et al*.* reported that the K31T mutation on hACE2 increases the binding affinity^25^, because the K31 in WT hACE2 (PDB ID: 2AJF) interacts only with Y442 of RBD-SARS-CoV-1, whereas T31 in the mutated hACE2 (PDB ID: 3D0G) interacts with two amino acids, Y442 and Y475.

### The common hot spot region in RBD-SARS-CoV-1 against hACE2 and SARS-CoV-1 antibodies

In order to narrow down the hot spot regions between hACE2 and RBD-SARS-CoV-1, we performed FMO calculations on four RBD-SARS-CoV-1/antibody complexes (Supplementary Table S14-S19). We summarized the FMO results in Fig. 1.

In RBD-SARS-CoV-1/80R (PDB ID: 2GHW) complex, the FMO results detected 30 amino acid pairs, which are summarized in Supplementary Table S14. The amino acid pairs were in agreement with the results previously reported by Hwang et al*.*^26^ When comparing the 30 amino acid pairs of this study with the previously reported results, it was confirmed that 17 of the 30 amino acid pairs are correlated: R426/Y53, T433/W226, N437/R162, Y440/D182, P470/D202, N479/D182, D480/R162, D480/S163, D480/N164, D480/R223, Y481/R223, Y484/Y102, T486/Y53, T487/Y53, T487/D99, G488/A33, and Y491/D99. In RBD-SARS-CoV-1/m395 (PDB ID: 2DD8) complex, the FMO results detected 18 amino acid pairs, which are summarized in Supplementary Table S15. The amino acid pairs that contributed to the stability of the complexes are well correlated with the published site-directed mutagenesis study, in which the T487 mutation does not significantly affect the neutralizing activity of the antibody^27^. The FMO results supported that T487S mutation would change only minor van der Waals interactions between T487 and _HC_Y32. In the RBD-SARS-CoV-1/S230 (PDB ID: 6NB6, 6NB7) complex, the FMO results detected 25 amino acid pairs, which are summarized in Supplementary Table S16-S18. The S230 binds to RBD-SARS-CoV-1 in different two states. The FMO results of state 1 are detailed in Supplementary Table S16, and those of the state 2 are mentioned in Supplementary Table S17-S18. In the RBD-SARS-CoV-1/F26G19 (PDB ID: 3BGF) complex, the FMO results detected 24 amino acid pairs, which are summarized in Supplementary Table S19.

In order to find common hot spot amino acids in RBD-SARS-CoV-1 against hACE2 and SARS-CoV-1 antibodies, we illustrated the FMO results with a 3D-SPIEs-based map. (see Fig. 1). All four antibodies (80R, m395, S230, and F26G19) and hACE2 have two common amino acids, R426 and T487, in RBD-SARS-CoV-1. Three of the four antibodies and hACE2 have four common amino acids, T486, G488, I489, and Y491, in RBD-SARS-CoV-1. Two of the four antibodies and hACE2 have two common amino acids, F483 and Q492, in RBD-SARS-CoV-1. Only S230 and hACE2 share four common amino acids, D463, N473, Y475, and Y442, in RBD-SARS-CoV-1. Only 80R and hACE2 share two common amino acids, Q479 and Y484, in RBD-SARS-CoV-1. Other interactions between antibodies and RBD-SARS-CoV-1 do not share interactions between hACE2 and RBD-SARS-CoV-1. Considering the possibility of mutation prediction in viruses by the FMO methods^28,29^, the evolutionary process of SARS-CoV-1 can be performed to elude neutralization of antibody by switching the unshared interactions between the antibody and hACE2 receptor.

According to the map, there are two hot spot regions between hACE2 and RBD-SARS-CoV-1 (See Fig. 1). The main hot spot region on hACE2 consists of D38, Y41, K353, D355, and several residues. The counter part of that on RBD-SARS-CoV-1 comprises R426, T486, T487, I489, Y491, and so on. The secondary hot spot region on hACE2 receptor consists of D30, K31, and several residues. The counter part of that on RBD-SARS-CoV-1 comprises Y442, D463, N473, N479, and so on. We found that SARS-CoV-1 antibodies focus on the main hot spot to block the formation of amino acid pairs between hACE2 and RBD-SARS-CoV-1.

### Hot spot region between hACE2 and RBD domains from SARS-CoV-1, HCoV-NL63, and SARS-CoV-2

Although the RBD of HCoV-NL63 does not share structural homology with the RBDs of SARS-CoV-1 and SARS-CoV-2, the three viruses recognize the same hACE2 receptor to invade host cells. In order to investigate the hot spot region between HCoV-NL63 and hACE2, we performed FMO calculations on the HCoV-NL63/hACE2 complex (PDB ID: 3KBH). The FMO results in which 23 amino acid pairs were detected are summarized in Supplementary Table S20. The FMO results were in agreement with the six amino acid pairs (hACE2/RBD-HCoV-NL63) previously reported by Wu et al.^30^: D30/S496, H34/G495, H34/S496, E37/Y498, M323/H586, and G354/G537.

In order to find amino acids in hot spot regions in the PPI interface between SARS-CoV-2 and hACE2, we performed FMO calculations on four SARS-CoV-2/hACE2 complexes (Supplementary Table S21-S24), the results of which are summarized in Fig. 1. The FMO results detected 37 amino acid pairs as the union of the amino acid pair members from all the four complexes. Among the 37 amino acid pairs, all four complexes have common interactions: 1) seven hydrogen bond interactions (hACE2/RBD): Q24/N487, E35/Q493, E37/Y505, D38/Y449, D38/G496, K353/F497, and G354/G502, 2) an amide-π interaction between G354 in hACE2 and Y505 in RBD-SARS-CoV-2, and 3) four electrostatic interactions (hACE2/RBD): K31/P491, G354/N501, D355/T500, and D355/G502. Three of the four complexes have common interactions between hACE2 and RBD-SARS-CoV-2: 1) six hydrogen bond interactions (hACE2/RBD): F28/Y489, D30/K417, K31/E484, K31/Q493, Y41/Q498, and Y83/N487, 2) a π–π interaction between Y83 in hACE2 F486 and in RBD-SARS-CoV-2, and 3) four electrostatic interactions (hACE2/RBD): S19/G476, S19/S477, Y41/N501, and R357/N501. Two of the four complexes have five common amino acid pairs in hACE2/RBD: D30/L455, D38/Q498, N330/N501, K353/Q498, and K353/G502.

To investigate the common hot spot region on hACE2 against RBDs from the three viruses, and vice versa, we illustrated the FMO results in Fig. 1. In the three viruses, all RBDs have common interactions with D30, K31, E37, K353, G354, and D355 in hACE2. SARS-CoV-1 and SARS-CoV-2 have common interactions with the S19, Q24, F28, E35, A36, D38, Y41, Q42, Y83, E329, N330, and R357 in hACE2. Only SARS-CoV-1 and hACE2 share interactions with E23, A25, F32, T324, Q325, G326, and F356, whereas only NL63-CoV and hACE2 share interactions with N33, M323, and F327. The common interactions between SARS-CoV1 and NL63-CoV were H34 and R393 in hACE2.

### Hot spot region in hACE2/RBD-SARS-CoV-2 integrated with 3D-SPIEs-based interaction map

**Figure 2:**
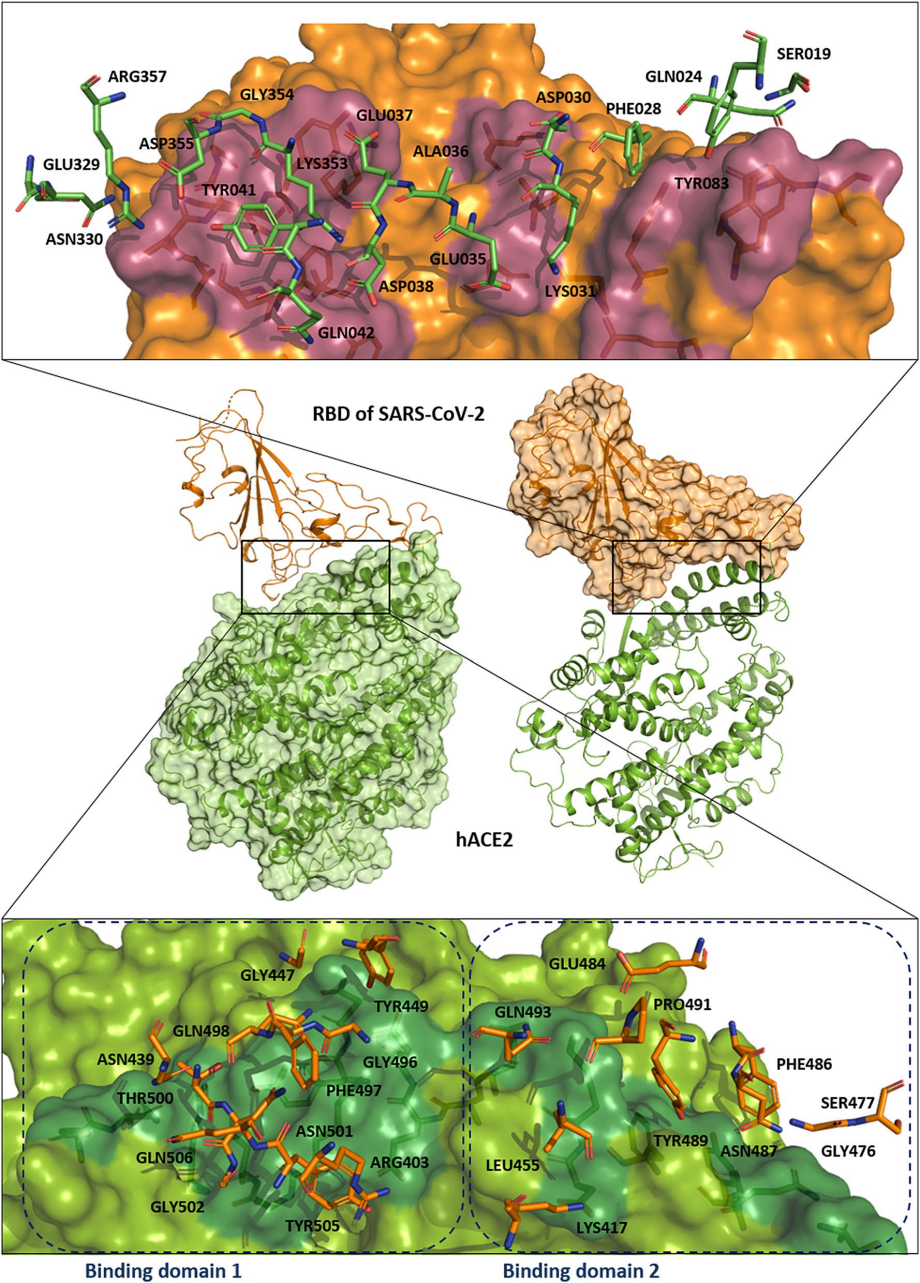
Two main hot spot regions between hACE2 and spike protein from SARS-CoV-2. The main first hot spot of hACE2 consists of D30 and K31, while the main second hot spot of hACE2 consists of E37, K353, G354, and D355. The hACE2 protein is represented by green ribbon and surface, while the RBD of spike protein from SARS-CoV-2 is represented by orange ribbon and surface (PDB ID: 6M0J). The key binding sites on surface from hACE2 are colored in dark green, while those from RBD-SARS-CoV-2 are colored in raspberry.

We created a 3D-SPIEs based interaction map to find the hot spot regions from the PPI information between hACE2 and RBD-SARS-CoV-2 (see Figs. 1 and 2). When comparing the interacting residues between hACE2 and RBD of the three viruses, there are two hot spot regions consisting of shallow grooves on the hACE2 receptor. The main hot spot is formed by E37, K353, G354 and D355. The secondary hot spot consists of D30 and K31. According to the map, the main hot spot is expected to be the most important hot spot between hACE2 and RBD-SARS-CoV-2. We observed that the secondary hot spot on hACE2 has interactions with K417, L455, E484, P491, and Q493 in RBD-SARS-CoV-2, whereas the main hot spot has interactions with R403, F497, Q498, T500, N501, G502, Y505, and Q506 in RBD-SARS-CoV-2. The results from the common hot spot region in SARS-CoV-1 antibodies supported the results that the main hot spot region was important for the PPI between RBD-SARS-CoV-1 and its antibodies. In the results of SARS-CoV-2 and its antibody (B38) summarized in Supplementary Table S25, the antibody had interactions with R403, Q498, N501, G502, and Y505 of RBD-SARS-CoV-2, which are the counterpart of the main hot spot. It can be used to develop antibodies and antiviral agents by using the information of the hot spot regions suggested in this work.

## Conclusions

Even though the FMO method was successfully applied to evaluate PPIs, analysis of biomolecular systems still requires huge computational costs. Here, we combined parameterized quantum chemical approaches (FMO-DFTB3/D/PCM) and the 3D-scattered pair interaction energies (3D-SPIEs) protocol to analyze PPIs between SARS-CoV-2 and hACE2 complex. The FMO-DFTB3/D/PCM/3D-SPIEs results also showed a qualitative correlation with site-directed mutagenesis results, such as the FMO-MP2/PCM/3D-SPIEs results in our earlier work^10^. The reliable inter-residue interaction energy calculation method, FMO-DFTB3/D/PCM/3D-SPIEs, would be a powerful tool for drug discovery and protein engineering in the future. Furthermore, the quantum–mechanical-level hot spot analysis results will provide new directions for antibody engineering and small-molecule development. The 3D-SPIEs-based map would provide valuable information for the discovery of anti-viral therapeutics that inhibit PPIs between the spike protein of SARS-CoV-2 and hACE2.

## Supplementary information

Supplementary Information 1.

## References

[1] Chan JF-W (2020). A familial cluster of pneumonia associated with the 2019 novel coronavirus indicating person-to-person transmission: a study of a family cluster. The Lancet.

[2] Huang C (2020). Clinical features of patients infected with 2019 novel coronavirus in Wuhan, China. The Lancet.

[3] Wrapp D (2020). Cryo-EM structure of the 2019-nCoV spike in the prefusion conformation. Science.

[4] Guo Y-R (2020). The origin, transmission and clinical therapies on coronavirus disease 2019 (COVID-19) outbreak–an update on the status. Mil. Med. Res..

[5] Li F (2016). Structure, function, and evolution of coronavirus spike proteins. Annu. Rev. Virol..

[6] Bosch BJ, van der Zee R, de Haan CA, Rottier PJ (2003). The coronavirus spike protein is a class I virus fusion protein: structural and functional characterization of the fusion core complex. J. Virol..

[7] Gaus M, Cui Q, Elstner M (2011). DFTB3: extension of the self-consistent-charge density-functional tight-binding method (SCC-DFTB). J. Chem. Theory Comput..

[8] Kitaura K, Ikeo E, Asada T, Nakano T, Uebayasi M (1999). Fragment molecular orbital method: an approximate computational method for large molecules. Chem. Phys. Lett..

[9] Nishimoto Y, Fedorov DG (2016). The fragment molecular orbital method combined with density-functional tight-binding and the polarizable continuum model. Phys. Chem. Chem. Phys..

[10] Lim H (2019). Investigation of protein-protein interactions and hot spot region between PD-1 and PD-L1 by fragment molecular orbital method. Sci. Rep..

[11] Jacobson MP, Friesner RA, Xiang Z, Honig B (2002). On the role of the crystal environment in determining protein side-chain conformations. J. Mol. Biol..

[12] Olsson MH, Søndergaard CR, Rostkowski M, Jensen JH (2011). PROPKA3: consistent treatment of internal and surface residues in empirical p K a predictions. J. Chem. Theory Comput..

[13] Alexeev Y, Mazanetz MP, Ichihara O, Fedorov D (2012). GAMESS as a free quantum-mechanical platform for drug research. Curr. Top. Med. Chem..

[14] Gaus M, Goez A, Elstner M (2013). Parametrization and benchmark of DFTB3 for organic molecules. J. Chem. Theory Comput..

[15] Gaus M, Lu X, Elstner M, Cui Q (2014). Parameterization of DFTB3/3OB for sulfur and phosphorus for chemical and biological applications. J. Chem. Theory Comput..

[16] Zhechkov L, Heine T, Patchkovskii S, Seifert G, Duarte HA (2005). An efficient a posteriori treatment for dispersion interaction in density-functional-based tight binding. J. Chem. Theory Comput..

[17] Rappé AK, Casewit CJ, Colwell K, Goddard WA, Skiff WM (1992). UFF, a full periodic table force field for molecular mechanics and molecular dynamics simulations. J. Am. Chem. Soc..

[18] Nakano T (2000). Fragment molecular orbital method: application to polypeptides. Chem. Phys. Lett..

[19] Nakano T (2002). Fragment molecular orbital method: use of approximate electrostatic potential. Chem. Phys. Lett..

[20] Heifetz A (2016). The Fragment molecular orbital method reveals new insight into the chemical nature of GPCR–ligand interactions. J. Chem. Inf. Model..

[21] Fedorov DG, Nagata T, Kitaura K (2012). Exploring chemistry with the fragment molecular orbital method. Phys. Chem. Chem. Phys..

[22] Wu K (2011). A virus-binding hot spot on human angiotensin-converting enzyme 2 is critical for binding of two different coronaviruses. J. Virol..

[23] Li W (2005). Receptor and viral determinants of SARS-coronavirus adaptation to human ACE2. EMBO J..

[24] Qu X-X (2005). Identification of two critical amino acid residues of the severe acute respiratory syndrome coronavirus spike protein for its variation in zoonotic tropism transition via a double substitution strategy. J. Biol. Chem..

[25] Wu K, Peng G, Wilken M, Geraghty RJ, Li F (2012). Mechanisms of host receptor adaptation by severe acute respiratory syndrome coronavirus. J. Biol. Chem..

[26] Hwang WC (2006). Structural basis of neutralization by a human anti-severe acute respiratory syndrome spike protein antibody, 80R. J. Biol. Chem..

[27] Prabakaran P (2006). Structure of severe acute respiratory syndrome coronavirus receptor-binding domain complexed with neutralizing antibody. J. Biol. Chem..

[28] Takematsu K (2009). Possibility of mutation prediction of influenza hemagglutinin by combination of hemadsorption experiment and quantum chemical calculation for antibody binding. J. Phys. Chem. B.

[29] Yoshioka A (2011). Prediction of probable mutations in influenza virus hemagglutinin protein based on large-scale ab initio fragment molecular orbital calculations. J. Mol. Graph. Model..

[30] Wu K, Li W, Peng G, Li F (2009). Crystal structure of NL63 respiratory coronavirus receptor-binding domain complexed with its human receptor. Proc. Natl. Acad. Sci..

