# Supplementary material for: Hot spot profiles of SARS-CoV-2 and human ACE2 receptor protein protein interaction obtained by density functional tight binding fragment molecular orbital method
Source: Sci Rep. 2020 Oct 8;10:16862. doi: 10.1038/s41598-020-73820-8 (PMC7544872; doi:10.1038/s41598-020-73820-8)
Supplement: Supplementary file 1 — Supplementary Information 1. [file 41598_2020_73820_MOESM1_ESM.pdf]

Supporting Information for

Hot Spot Profiles of SARS-CoV-2 and Human ACE2 Receptor Protein Protein Interaction Obtained by Density Functional Tight Binding Fragment Molecular Orbital Method

Hocheol Lim[a,b,c,*], Ayoung Baek[c,*], Jongwan Kim[a,c,*], Min Sung Kim[a,c], Jiaxin Liu[a], Ky-Youb Nam[d], JeongHyeok Yoon[d], and Kyoung Tai No[a,b,c].

[a] Department of Biotechnology, Yonsei University, Seoul, Republic of Korea

[b] The Interdisciplinary Graduate program in Integrative Biotechnology & Translational Medicine, Yonsei University, Incheon, Republic of Korea

[c] Bioinformatics and Molecular Design Research Center (BMDRC), Incheon, Republic of Korea

[d] Pharos I&BT Co., Ltd., Anyang-si, Gyeonggi-do, Republic of Korea

[*] Co-first authors

Corresponding author: Kyoung Tai No (ktno@yonsei.ac.kr)

Supporting Information for 'Hot Spot Profiles of SARS-CoV-2 and Human ACE2 Receptor Protein-Protein Interaction Obtained by Density Functional Tight-Binding Fragment Molecular Orbital Method' includes Table S1 for mutation information in hACE2/RBD-SARS-CoV-1 complexes. It includes Table S2 – S13 for PIEDA results between hACE2 and SARS-CoV-1. It includes Table S14 – S19 for PIEDA results between SARS-CoV-1 and four antibodies (80R, m395, S230, and F26G19). It includes Table S20 for PIEDA results between hACE2 and HCoV-NL63. It includes Table S21 – S24 for PIEDA results between hACE2 and SARS-CoV-2. It includes Table S25 for PIEDA results between SARS-CoV-2 and its antibody (B38).

Table S1 Mutation information of hACE2/RBD-SARS-CoV-1 complexes

PDB ID	hACE2	RBD-SARS-CoV-1
3D0G	I21T, Q24L, D30E, K31T, H34Y, E37Q, D38E, F40S, L45V	-
3D0H	I21T, Q24L, D30E, K31T, H34Y, E37Q, D38E, F40S, L45V	N479K, T487S
3D0I	I21T, Q24L, D30E, K31T, H34Y, E37Q, D38E, F40S, L45V	N479R, D480G, T487S
3SCI	-	Y442F, L472F
3SCJ	-	L472P, N479R, D480G
3SCK	I21T, Q24L, D30E, K31T, H34Y, E37Q, D38E, F40S, L45V, M82T	L472P, N479R, D480G
3SCL	I21T, Q24L, D30E, K31T, H34Y, E37Q, F40S, L45V, M82T	-

Table S2 The PIEDA Analysis of hACE2/RBD-SARS-CoV-1 complex (PDB ID: 2AJF)

hACE2	RBD-SARS-CoV-1	PIE	ΔE^{es}	ΔE^{ex}	ΔE^{ct+mix}	ΔE^{di}	ΔG^{sol}
SER019	ASP463	-24.264	-44.053	0.000	-0.001	-0.846	20.635
GLN024	ASN473	-8.059	-5.982	0.000	-0.092	-0.632	-1.353
LYS031	TYR442	-3.760	0.648	0.000	0.000	-2.878	-1.529
	TRP476	-6.050	-0.114	0.000	0.000	-0.222	-5.714
GLU037	TYR491	-8.464	-8.036	0.000	-0.194	-1.243	1.009
ASP038	TYR436	-23.299	-22.435	0.000	-2.141	0.777	0.500
	GLY482	-11.349	-8.118	0.000	-0.010	-0.375	-2.847
	TYR484	-8.535	-8.137	0.000	-0.007	-0.623	0.232
TYR041	THR487	-3.851	-1.225	0.000	-0.051	-2.728	0.153
GLN042	TYR436	-4.131	-3.537	0.000	-0.017	-0.906	0.328
TYR083	ASN473	-6.619	-6.412	0.000	-0.128	-0.934	0.855
GLN325	ARG426	-5.402	-2.288	0.000	-0.017	-0.996	-2.101
GLU329	ARG426	-58.656	-89.351	0.000	-1.362	-0.095	32.151
	GLN492	-3.211	-4.578	0.000	0.000	-0.092	1.459
ASN330	THR487	-5.344	-4.509	0.000	0.307	-0.294	-0.848
LYS353	PHE483	-15.594	-16.454	0.000	-0.068	-0.813	1.740
	GLY488	-3.517	-2.664	0.000	0.000	-0.451	-0.403
	GLN492	-3.552	-5.573	0.000	0.000	-0.316	2.337
GLY354	THR487	-5.492	-4.593	0.000	0.009	-0.591	-0.317
	GLY488	-5.086	-4.451	0.000	0.236	-0.232	-0.638
	TYR491	-6.448	-4.118	0.000	0.088	-2.550	0.133
ASP355	THR486	-10.714	-10.059	0.000	-0.005	-0.650	0.001
ARG357	THR487	-7.745	-8.528	0.000	-0.004	-0.474	1.260

All energies are in kcal/mol.

The calculation has FMO-DFTB3/D/PCM level

Table S3 The PIEDA Analysis of hACE2/RBD-SARS-CoV-1 complex (PDB ID: 3D0G)

hACE2	RBD-SARS-CoV-1	PIE	ΔE^{es}	ΔE^{ex}	ΔE^{ct+mix}	ΔE^{di}	ΔG^{sol}
SER019	ASP463	-46.679	-74.801	0.000	-0.250	-1.168	29.541
LEU024	ASP463	-3.322	-4.239	0.000	0.000	-0.579	1.496
	ASN473	-3.820	-1.106	0.000	-0.034	-2.118	-0.561
PHE028	TYR475	-4.050	-1.559	0.000	-0.018	-2.781	0.308
GLU030	VAL404	-3.391	-4.471	0.000	0.000	-0.144	1.224
	TYR442	-5.040	-4.914	0.000	-0.027	-1.260	1.160
THR031	TYR442	-5.205	-2.413	0.000	-0.933	-1.999	0.140
PHE032	TYR442	-3.060	-2.495	0.000	-0.014	-0.397	-0.155
TYR034	ASN479	-4.055	-2.560	0.000	0.033	-1.606	0.078
GLU038	TYR436	-15.140	-13.781	0.000	-1.738	-0.868	1.247
	GLY482	-10.668	-3.707	0.000	-0.324	-0.794	-5.844
	TYR484	-5.617	-5.169	0.000	-0.008	-0.717	0.277
TYR041	TYR484	-3.624	-0.064	0.000	0.129	-3.665	-0.024
	THR486	-3.487	-2.727	0.000	-0.520	-0.252	0.012
	THR487	-4.209	-1.745	0.000	-0.047	-2.550	0.133
TYR083	ASN473	-6.485	-6.013	0.000	-0.448	-0.577	0.552
GLN325	ARG426	-10.541	-6.591	0.000	-0.015	-0.727	-3.207
GLU329	ARG426	-33.474	-70.058	0.000	-0.071	-0.708	37.363
	GLN492	-3.875	-5.262	0.000	0.000	-0.084	1.471
ASN330	THR487	-4.287	-3.082	0.000	0.008	-0.657	-0.557
LYS353	PHE483	-20.190	-20.771	0.000	-0.176	-0.841	1.597
	GLY488	-3.365	-2.511	0.000	0.000	-0.390	-0.464
	GLN492	-4.523	-7.596	0.000	0.000	-0.286	3.358
GLY354	THR487	-5.515	-4.541	0.000	0.009	-0.592	-0.391
	GLY488	-4.559	-3.372	0.000	-0.126	-0.383	-0.678
	TYR491	-5.637	-2.266	0.000	0.086	-2.681	-0.776
ASP355	THR486	-10.218	-9.746	0.000	-0.002	-0.578	0.108
ARG357	THR487	-10.287	-11.570	0.000	-0.020	-0.562	1.864

All energies are in kcal/mol.

The calculation has FMO-DFTB3/D/PCM level

Table S4 The PIEDA Analysis of hACE2/RBD-SARS-CoV-1 complex (PDB ID: 3D0H)

hACE2	RBD-SARS-CoV-1	PIE	ΔE^{es}	ΔE^{ex}	ΔE^{ct+mix}	ΔE^{di}	ΔG^{sol}
SER019	ASP463	-53.839	-78.486	0.000	0.253	-1.094	25.488
LEU024	ASP463	-5.106	-4.695	0.000	-0.001	-0.523	0.113
	ASN473	-3.141	-1.297	0.000	0.022	-1.641	-0.225
PHE028	TYR475	-4.449	-1.985	0.000	-0.027	-2.695	0.258
GLU030	TYR442	-5.495	-5.174	0.000	-0.001	-0.895	0.575
THR031	TYR442	-5.427	-3.100	0.000	-0.780	-1.702	0.156
PHE032	LYS479	-6.105	-7.781	0.000	0.000	-0.161	1.838
GLU035	LYS479	-57.839	-85.583	0.000	-0.297	-2.864	30.905
ALA036	LYS479	-12.827	-13.917	0.000	-0.001	-0.292	1.383
GLN037	TYR491	-4.651	-2.461	0.000	-0.272	-1.007	-0.911
GLU038	LYS479	-38.465	-49.751	0.000	-0.061	-1.151	12.498
	GLY482	-7.156	-0.668	0.000	-0.132	-0.834	-5.522
TYR041	TYR484	-3.793	-0.515	0.000	0.351	-3.580	-0.049
	SER487	-3.184	-1.502	0.000	-0.017	-1.812	0.146
TYR083	ASN473	-3.856	-4.273	0.000	-0.068	-0.598	1.083
GLN325	ARG426	-7.380	-5.572	0.000	-0.006	-0.565	-1.237
GLU329	ARG426	-24.798	-66.217	0.000	0.000	-0.590	42.009
ASN330	SER487	-5.068	-3.525	0.000	0.005	-0.404	-1.144
LYS353	PHE483	-15.940	-18.091	0.000	0.105	-0.648	2.694
	GLN492	-7.034	-8.602	0.000	0.000	-0.328	1.896
GLY354	SER487	-5.095	-4.242	0.000	-0.013	-0.533	-0.308
	GLY488	-5.000	-5.426	0.000	1.430	-0.096	-0.908
	TYR491	-6.650	-4.110	0.000	0.165	-2.851	0.147
ASP355	THR486	-10.676	-9.810	0.000	-0.003	-0.604	-0.259
	GLY488	-3.217	-2.024	0.000	0.043	-1.904	0.668
ARG357	SER487	-6.892	-8.482	0.000	-0.002	-0.426	2.018

All energies are in kcal/mol.

The calculation has FMO-DFTB3/D/PCM level

Table S5 The PIEDA Analysis of hACE2/RBD-SARS-CoV-1 complex (PDB ID: 3D0I)

hACE2	RBD-SARS-CoV-1	PIE	ΔE^{es}	ΔE^{ex}	ΔE^{ct+mix}	ΔE^{di}	ΔG^{sol}
SER019	ASP463	-27.879	-51.523	0.000	0.019	-1.716	25.341
LEU024	ASP463	-6.504	-5.243	0.000	-0.002	-0.791	-0.468
	ASN473	-4.420	-2.626	0.000	0.073	-1.493	-0.374
PHE028	TYR475	-4.685	-1.889	0.000	-0.024	-2.986	0.214
GLU030	TYR442	-5.646	-5.131	0.000	-0.017	-1.131	0.633
THR031	TYR442	-5.280	-2.739	0.000	-0.987	-1.778	0.223
PHE032	TYR442	-3.204	-2.526	0.000	-0.011	-0.395	-0.271
	ARG479	-7.078	-8.997	0.000	0.000	-0.212	2.132
GLU035	ARG479	-56.021	-83.728	0.000	-0.603	-1.047	29.358
ALA036	ARG479	-12.188	-13.246	0.000	0.094	-0.411	1.375
GLU038	ARG479	-32.987	-45.878	0.000	0.341	-0.868	13.417
	GLY482	-5.492	0.749	0.000	-0.118	-0.816	-5.307
TYR041	TYR484	-3.740	-0.642	0.000	0.560	-3.594	-0.063
	SER487	-3.223	-1.537	0.000	-0.014	-1.805	0.133
GLN042	TYR484	-3.168	-2.661	0.000	0.250	-1.064	0.307
TYR083	ASN473	-5.357	-5.259	0.000	-0.276	-0.930	1.109
GLU329	ARG426	-25.774	-64.845	0.000	0.000	-0.510	39.582
ASN330	SER487	-3.859	-2.654	0.000	0.006	-0.598	-0.614
LYS353	TYR436	-3.170	-0.809	0.000	0.000	-0.372	-1.989
	GLY482	-3.674	-8.803	0.000	-0.015	-0.786	5.930
	PHE483	-18.132	-18.284	0.000	-0.090	-0.966	1.208
	TYR491	-7.217	2.968	0.000	0.062	-4.620	-5.627
	GLN492	-4.595	-8.054	0.000	0.000	-0.302	3.761
GLY354	SER487	-4.346	-3.544	0.000	-0.025	-0.536	-0.242
	GLY488	-5.560	-5.500	0.000	0.939	-0.158	-0.842
	TYR491	-5.580	-2.168	0.000	0.185	-2.724	-0.873
ASP355	THR486	-11.673	-10.007	0.000	-0.006	-0.572	-1.088
ARG357	SER487	-8.253	-9.263	0.000	-0.011	-0.497	1.518
ARG393	TYR491	-5.860	0.118	0.000	-0.002	-0.748	-5.229

All energies are in kcal/mol.

The calculation has FMO-DFTB3/D/PCM level

Table S6 The PIEDA Analysis of hACE2/RBD-SARS-CoV-1 complex (PDB ID: 3SCI)

hACE2	RBD-SARS-CoV-1	PIE	ΔE^{es}	ΔE^{ex}	ΔE^{ct+mix}	ΔE^{di}	ΔG^{sol}
SER019	ASP463	-48.394	-67.252	0.000	-0.123	-1.047	20.029
GLN024	ASN473	-7.748	-7.876	0.000	-0.008	-0.952	1.087
PHE028	TYR475	-4.459	-1.380	0.000	-0.034	-3.187	0.141
LYS031	PRO477	-3.842	-5.359	0.000	0.000	-0.108	1.626
HIS034	LYS390	-3.907	-5.959	0.000	-0.024	-0.670	2.746
GLU037	TYR491	-13.615	-11.461	0.000	-0.100	-1.397	-0.657
ASP038	TYR436	-20.480	-20.039	0.000	-1.866	0.773	0.652
	GLY482	-10.000	-8.108	0.000	-0.039	-0.470	-1.383
	TYR484	-11.406	-9.931	0.000	-0.044	-0.809	-0.623
TYR041	TYR484	-3.048	0.107	0.000	0.168	-3.391	0.069
	THR487	-4.632	-2.466	0.000	-0.021	-2.201	0.056
TYR083	ASN473	-5.293	-4.523	0.000	-0.174	-1.011	0.415
GLN325	ARG426	-9.537	-5.238	0.000	-0.004	-0.754	-3.541
GLU329	ARG426	-64.230	-93.224	0.000	-1.815	0.401	30.409
	GLN492	-4.768	-6.337	0.000	0.000	-0.138	1.707
ASN330	THR487	-3.710	-1.981	0.000	0.061	-0.765	-1.026
LYS353	GLY482	-6.892	-12.570	0.000	0.163	-0.551	6.066
	PHE483	-23.365	-23.529	0.000	-0.163	-0.348	0.675
	GLN492	-4.939	-7.631	0.000	0.000	-0.273	2.965
GLY354	THR487	-5.511	-4.834	0.000	0.051	-0.626	-0.101
	GLY488	-4.047	-2.950	0.000	-0.149	-0.329	-0.619
	TYR491	-6.564	-4.116	0.000	0.094	-2.863	0.321
ASP355	THR486	-9.877	-9.824	0.000	-0.002	-0.547	0.496
	GLY488	-3.495	-3.461	0.000	-0.004	-1.666	1.636
	TYR491	-3.416	-2.324	0.000	0.004	-0.495	-0.600
ARG357	THR487	-11.956	-11.803	0.000	-0.033	-0.551	0.432

All energies are in kcal/mol.

The calculation has FMO-DFTB3/D/PCM level

Table S7 The PIEDA Analysis of hACE2/RBD-SARS-CoV-1 complex (PDB ID: 3SCJ)

hACE2	RBD-SARS-CoV-1	PIE	ΔE^{es}	ΔE^{ex}	ΔE^{ct+mix}	ΔE^{di}	ΔG^{sol}
SER019	ASP463	-53.305	-71.088	0.000	0.260	-0.789	18.312
	GLY464	-8.377	-12.386	0.000	-0.017	-0.166	4.191
GLN024	ASP463	-6.302	-10.538	0.000	0.000	-0.393	4.629
ALA025	PRO462	-3.116	-3.297	0.000	0.006	-0.327	0.503
	ASN473	-3.908	-3.543	0.000	0.000	-0.354	-0.011
PHE028	TYR475	-5.402	-2.284	0.000	-0.077	-3.167	0.126
ASP030	TYR442	-6.460	-7.747	0.000	-0.064	-1.312	2.663
LYS031	TYR442	-4.130	-0.154	0.000	0.218	-2.638	-1.556
PHE032	ARG479	-8.321	-8.900	0.000	0.000	-0.220	0.800
GLU035	ARG479	-36.859	-56.502	0.000	0.165	-3.291	22.769
ALA036	ARG479	-9.715	-11.989	0.000	0.005	-0.340	2.610
GLU037	TYR491	-8.303	-7.029	0.000	-0.170	-1.688	0.584
ASP038	TYR436	-3.547	-5.877	0.000	-0.305	-1.431	4.067
	ARG479	-52.576	-69.412	0.000	-0.009	-0.196	17.042
TYR041	THR487	-4.637	-1.958	0.000	-0.053	-2.735	0.109
GLN325	ARG426	-12.758	-7.330	0.000	-0.035	-0.692	-4.701
GLU329	ARG426	-64.771	-95.287	0.000	-1.651	0.486	31.682
	GLN492	-3.994	-5.968	0.000	0.000	-0.124	2.097
ASN330	THR487	-5.225	-3.826	0.000	0.132	-0.566	-0.964
LYS353	GLY482	-8.159	-10.718	0.000	0.038	-0.657	3.179
	PHE483	-21.302	-20.921	0.000	-0.274	-0.508	0.402
	GLY488	-3.110	-2.540	0.000	0.000	-0.447	-0.123
	GLN492	-5.929	-8.142	0.000	0.000	-0.283	2.495
GLY354	THR487	-5.076	-4.197	0.000	-0.025	-0.648	-0.206
	GLY488	-4.549	-3.423	0.000	-0.139	-0.390	-0.597
	TYR491	-6.314	-4.356	0.000	0.183	-2.315	0.174
ASP355	THR486	-10.587	-10.169	0.000	-0.004	-0.631	0.216
ARG357	THR487	-7.979	-8.733	0.000	-0.002	-0.503	1.260

All energies are in kcal/mol.

The calculation has FMO-DFTB3/D/PCM level

Table S8 The PIEDA Analysis of hACE2/RBD-SARS-CoV-1 complex (PDB ID: 3SCK)

hACE2	RBD-SARS-CoV-1	PIE	ΔE^{es}	ΔE^{ex}	ΔE^{ct+mix}	ΔE^{di}	ΔG^{sol}
SER019	ASP463	-29.655	-48.450	0.000	0.004	-1.461	20.253
GLU023	PRO462	-3.435	-5.093	0.000	-0.001	-0.520	2.178
LEU024	ASP463	-3.581	-3.563	0.000	0.000	-0.479	0.460
	ASN473	-4.192	-1.489	0.000	-0.043	-2.297	-0.363
PHE028	TYR475	-3.832	-1.290	0.000	-0.010	-2.859	0.327
THR031	TYR442	-5.174	-3.492	0.000	0.148	-1.361	-0.469
PHE032	ARG479	-7.880	-9.703	0.000	0.000	-0.270	2.093
GLU035	ARG479	-60.192	-83.017	0.000	-0.899	-2.599	26.323
ALA036	ARG479	-11.879	-12.450	0.000	0.003	-0.359	0.927
GLU038	ARG479	-38.392	-48.803	0.000	0.005	-0.631	11.037
TYR041	TYR484	-3.712	-0.583	0.000	0.336	-3.522	0.058
GLN042	TYR484	-3.359	-3.653	0.000	0.429	-0.873	0.738
TYR083	ASN473	-7.449	-7.242	0.000	-0.180	-0.359	0.331
GLN325	ARG426	-14.005	-10.833	0.000	0.156	-0.213	-3.115
GLU329	ARG426	-43.318	-77.822	0.000	-0.098	-1.042	35.643
ASN330	THR487	-4.337	-3.269	0.000	0.353	-0.081	-1.340
LYS353	GLY482	-11.274	-14.857	0.000	0.156	-0.301	3.728
	PHE483	-19.858	-18.797	0.000	-0.061	-0.539	-0.461
	TYR491	-6.337	3.950	0.000	-0.070	-4.568	-5.648
	GLN492	-6.252	-8.604	0.000	0.000	-0.343	2.696
GLY354	THR487	-4.831	-3.922	0.000	-0.014	-0.633	-0.262
	GLY488	-4.824	-3.655	0.000	-0.091	-0.402	-0.676
	TYR491	-5.988	-2.511	0.000	0.140	-2.633	-0.984
ASP355	GLY488	-3.036	-2.450	0.000	-0.047	-1.828	1.289
ARG357	THR487	-7.876	-8.788	0.000	-0.005	-0.542	1.458
ARG393	TYR491	-5.583	0.214	0.000	0.000	-0.482	-5.315

All energies are in kcal/mol.

The calculation has FMO-DFTB3/D/PCM level

Table S9 The PIEDA Analysis of hACE2/RBD-SARS-CoV-1 complex (PDB ID: 3SCL)

hACE2	RBD-SARS-CoV-1	PIE	ΔE^{es}	ΔE^{ex}	ΔE^{ct+mix}	ΔE^{di}	ΔG^{sol}
SER019	ASP463	-48.841	-76.109	0.000	-0.045	-1.315	28.628
GLU023	PRO462	-3.207	-4.805	0.000	0.000	-0.299	1.896
LEU024	ASP463	-4.072	-4.857	0.000	0.000	-0.464	1.250
	ASN473	-4.220	-1.868	0.000	-0.081	-1.826	-0.445
PHE028	TYR475	-4.254	-1.820	0.000	-0.031	-2.676	0.273
GLU030	VAL404	-3.042	-4.994	0.000	0.000	-0.154	2.106
THR031	TYR442	-5.370	-3.383	0.000	-0.084	-1.527	-0.376
GLU035	ASN479	-7.392	-0.892	0.000	0.000	-0.340	-6.160
GLU038	TYR436	-12.584	-12.429	0.000	-1.205	-0.860	1.910
	GLY482	-4.157	0.090	0.000	-0.091	-0.839	-3.316
	TYR484	-5.680	-5.878	0.000	-0.007	-0.683	0.888
TYR041	THR487	-4.640	-2.108	0.000	-0.073	-2.599	0.140
TYR083	ASN473	-7.327	-6.586	0.000	-0.491	-0.521	0.272
GLN325	ARG426	-9.577	-4.769	0.000	-0.012	-0.801	-3.996
GLU329	ARG426	-53.276	-86.813	0.000	-1.698	0.000	35.235
ASN330	THR487	-3.265	-1.842	0.000	0.014	-0.697	-0.741
LYS353	GLY482	-9.575	-13.850	0.000	0.188	-0.462	4.549
	PHE483	-22.597	-22.750	0.000	0.004	-0.302	0.451
	GLY488	-3.404	-2.590	0.000	0.000	-0.457	-0.357
	GLN492	-5.496	-8.326	0.000	0.000	-0.313	3.142
GLY354	THR487	-5.472	-4.505	0.000	0.015	-0.618	-0.365
	GLY488	-4.623	-3.886	0.000	0.278	-0.245	-0.771
	TYR491	-6.765	-4.490	0.000	0.174	-2.586	0.138
ASP355	THR486	-9.847	-8.365	0.000	0.000	-0.560	-0.922
ARG357	THR487	-9.014	-10.261	0.000	-0.013	-0.559	1.819

All energies are in kcal/mol.

The calculation has FMO-DFTB3/D/PCM level

Table S10 The PIEDA Analysis of hACE2/RBD-SARS-CoV-1 complex (PDB ID: 6ACG)

hACE2	RBD-SARS-CoV-1	PIE	ΔE^{es}	ΔE^{ex}	ΔE^{ct+mix}	ΔE^{di}	ΔG^{sol}
SER019	ASP463	-34.428	-62.364	0.000	-0.020	-1.325	29.281
GLN024	ASN473	-3.804	-0.907	0.000	0.082	-3.001	0.022
ASP030	TYR442	-4.145	-4.924	0.000	0.073	-1.761	2.467
GLU035	ASN479	-5.113	-2.044	0.000	0.000	-0.533	-2.535
ASP038	TYR436	-6.150	-4.587	0.000	0.078	-1.262	-0.379
	GLY482	-17.318	-17.225	0.000	-0.071	-0.546	0.525
	TYR484	-3.837	-2.905	0.000	0.000	-0.397	-0.535
TYR041	THR487	-3.514	-1.694	0.000	-0.008	-1.859	0.047
GLN042	TYR436	-7.109	-6.954	0.000	-0.200	-0.211	0.255
	TYR484	-5.286	-4.456	0.000	0.010	-1.263	0.423
GLN325	ARG426	-8.675	0.264	0.000	0.207	-1.099	-8.046
	GLN492	-6.015	-5.068	0.000	-0.003	-0.508	-0.436
GLY326	ILE489	-7.447	-7.207	0.000	0.141	-0.724	0.342
PHE327	GLY488	-3.208	-2.903	0.000	0.000	-0.238	-0.068
GLU329	GLY488	-3.061	-0.845	0.000	0.000	-0.191	-2.025
ASN330	THR487	-5.955	-4.521	0.000	0.140	-0.587	-0.987
LYS353	PHE483	-27.322	-26.802	0.000	0.263	-0.111	-0.672
GLY354	THR487	-4.049	-3.266	0.000	-0.003	-0.586	-0.194
	TYR491	-6.285	-5.307	0.000	0.005	-1.524	0.540
ASP355	THR486	-6.301	-5.671	0.000	-0.001	-0.382	-0.248
	GLY488	-4.933	-4.521	0.000	0.000	-0.545	0.133
	GLY490	-4.054	-6.027	0.000	0.000	-0.153	2.126
ARG357	THR487	-6.470	-6.999	0.000	0.000	-0.252	0.781
ARG393	TYR491	-3.666	-2.026	0.000	-0.023	-0.747	-0.869

All energies are in kcal/mol.

The calculation has FMO-DFTB3/D/PCM level

Table S11 The PIEDA Analysis of hACE2/RBD-SARS-CoV-1 complex (PDB ID: 6ACJ)

hACE2	RBD-SARS-CoV-1	PIE	ΔE^{es}	ΔE^{ex}	ΔE^{ct+mix}	ΔE^{di}	ΔG^{sol}
SER019	ASP463	-30.977	-59.561	0.000	-0.068	-0.919	29.571
GLN024	ASN473	-5.418	-2.686	0.000	-0.011	-1.991	-0.730
PHE028	TYR475	-4.465	-1.156	0.000	-0.072	-3.195	-0.042
LYS031	TRP476	-4.258	5.181	0.000	-0.003	-0.955	-8.481
	PRO477	-7.609	-9.828	0.000	0.000	-0.230	2.449
	ASN479	-5.009	-3.821	0.000	-0.001	-0.588	-0.599
GLU037	GLY482	-3.263	-0.087	0.000	0.000	-0.171	-3.005
ASP038	TYR436	-14.929	-13.677	0.000	-0.475	0.186	-0.963
	GLY482	-19.021	-17.236	0.000	-0.035	-0.589	-1.161
	TYR484	-3.822	-2.626	0.000	0.000	-0.340	-0.856
TYR041	TYR484	-3.006	-0.729	0.000	0.074	-2.337	-0.015
TYR083	ASN473	-4.811	-4.035	0.000	0.056	-0.227	-0.605
GLN325	ARG426	-3.022	1.133	0.000	-0.132	-1.419	-2.605
	ILE489	-3.285	-0.939	0.000	0.161	-2.648	0.141
GLY326	ILE489	-5.677	-5.008	0.000	0.002	-0.602	-0.069
GLU329	THR485	-3.094	-5.139	0.000	0.000	-0.269	2.314
	GLN492	-4.766	-6.794	0.000	0.000	-0.175	2.203
LYS353	PHE483	-14.397	-13.880	0.000	0.823	-2.150	0.810
	GLN492	-3.933	-4.046	0.000	0.000	-0.201	0.315
GLY354	THR487	-6.125	-5.396	0.000	0.215	-0.690	-0.254
ASP355	THR486	-11.994	-10.532	0.000	-0.027	-0.646	-0.789
	GLY488	-5.843	-4.466	0.000	0.002	-1.439	0.059
	TYR491	-4.086	-3.351	0.000	-0.006	-0.357	-0.371
ARG357	THR487	-9.557	-9.845	0.000	0.000	-0.489	0.777

All energies are in kcal/mol.

The calculation has FMO-DFTB3/D/PCM level

Table S12 The PIEDA Analysis of hACE2/RBD-SARS-CoV-1 complex (PDB ID: 6ACK)

hACE2	RBD-SARS-CoV-1	PIE	ΔE^{es}	ΔE^{ex}	ΔE^{ct+mix}	ΔE^{di}	ΔG^{sol}
SER019	ASP463	-38.308	-70.039	0.000	-0.022	-1.167	32.920
GLN024	ASN473	-3.439	-0.610	0.000	0.045	-2.804	-0.070
LYS031	TYR475	-11.998	4.385	0.000	-0.277	-4.573	-11.532
GLU035	ASN479	-4.912	-0.157	0.000	0.000	-0.604	-4.151
ASP038	TYR436	-9.712	-8.738	0.000	-0.039	-1.251	0.315
	GLY482	-11.160	-10.709	0.000	-0.001	-0.372	-0.077
	TYR484	-5.307	-5.029	0.000	-0.001	-0.479	0.203
GLN042	TYR436	-7.255	-7.115	0.000	-0.398	-0.227	0.485
	TYR484	-4.223	-3.006	0.000	-0.059	-1.357	0.199
THR324	ILE489	-3.551	-1.681	0.000	0.020	-1.041	-0.849
GLN325	ARG426	-13.136	-3.547	0.000	-0.381	-0.385	-8.823
GLY326	THR487	-4.701	-4.598	0.000	-0.030	-0.442	0.370
	ILE489	-3.438	-2.942	0.000	0.000	-0.336	-0.161
GLU329	ARG426	-17.012	-52.012	0.000	0.000	-0.320	35.319
	THR485	-4.088	-6.160	0.000	-0.002	-0.377	2.451
	THR486	-8.042	-4.664	0.000	-0.002	-0.827	-2.549
ASN330	THR486	-4.664	-2.422	0.000	-0.097	-1.604	-0.542
LYS353	TYR436	-3.950	-3.271	0.000	-0.010	-1.060	0.390
	PHE483	-20.791	-23.080	0.000	0.694	-0.006	1.602
GLY354	THR487	-4.851	-4.065	0.000	0.102	-0.600	-0.288
	TYR491	-3.559	-2.795	0.000	0.591	-0.985	-0.371
ASP355	THR486	-20.296	-17.399	0.000	-0.822	-0.406	-1.671
PHE356	THR487	-3.691	-3.285	0.000	-0.007	-0.270	-0.129
ARG357	THR487	-3.936	-5.132	0.000	0.000	-0.663	1.860

All energies are in kcal/mol.

The calculation has FMO-DFTB3/D/PCM level

Table S13 The PIEDA Analysis of hACE2/RBD-SARS-CoV-1 complex (PDB ID: 6CS2)

hACE2	RBD-SARS-CoV-1	PIE	ΔE^{es}	ΔE^{ex}	ΔE^{ct+mix}	ΔE^{di}	ΔG^{sol}
SER019	ASP463	-36.382	-49.111	0.000	0.396	-0.043	12.376
	GLY464	-11.745	-16.461	0.000	0.097	-0.527	5.145
GLN024	ASP463	-9.385	-10.669	0.000	-0.027	-1.406	2.717
ALA025	ASN473	-6.161	-5.158	0.000	0.014	-0.650	-0.368
PHE028	TYR475	-5.443	-2.013	0.000	0.064	-3.577	0.084
ASP030	LEU443	-3.237	2.042	0.000	-0.008	-0.932	-4.338
	ASN479	-4.612	-3.541	0.000	0.000	-0.158	-0.913
LYS031	TYR442	-3.112	1.151	0.000	-0.020	-2.998	-1.245
	TRP476	-4.516	4.349	0.000	-0.003	-1.022	-7.840
	PRO477	-7.871	-8.851	0.000	0.000	-0.252	1.232
	ASN479	-4.213	-8.355	0.000	-0.023	-1.009	5.174
GLU035	ASN479	-4.925	2.400	0.000	0.000	-0.670	-6.656
ASP038	TYR436	-16.513	-12.763	0.000	-0.559	-1.110	-2.081
	GLY482	-10.864	-9.032	0.000	0.000	-0.280	-1.551
TYR083	ASN473	-4.466	-3.164	0.000	-0.048	-1.616	0.362
GLU329	ARG426	-85.430	-109.494	0.000	-4.206	1.633	26.637
ASN330	THR487	-3.151	-1.722	0.000	0.500	-0.466	-1.462
LYS353	PHE483	-12.966	-14.278	0.000	0.299	-0.764	1.777
	TYR484	-3.074	0.800	0.000	-0.001	-1.682	-2.191
GLY354	THR487	-5.060	-4.183	0.000	-0.010	-0.732	-0.134
	GLY488	-5.443	-4.658	0.000	-0.088	-0.267	-0.431
	TYR491	-3.946	-1.947	0.000	0.330	-1.758	-0.570
ASP355	THR486	-5.187	-5.724	0.000	0.000	-0.483	1.019
	TYR491	-3.247	-3.793	0.000	0.095	-0.629	1.080
ARG357	THR487	-9.003	-9.503	0.000	-0.001	-0.390	0.892

All energies are in kcal/mol.

The calculation has FMO-DFTB3/D/PCM level

Table S14 The PIEDA Analysis of RBD-SARS-CoV-1/80R complex (PDB ID: 2GHW)

RBD-SARS-CoV-1	80R	PIE	ΔE^{es}	ΔE^{ex}	ΔE^{ct+mix}	ΔE^{di}	ΔG^{sol}
ARG426	TYR053	-8.163	-2.739	0.000	0.031	-1.510	-3.946
	ASP054	-39.143	-60.806	0.000	-0.001	-0.397	22.061
SER432	TYR059	-3.625	-0.549	0.000	0.079	-2.126	-1.029
THR433	TRP226	-4.086	1.104	0.000	-0.263	-4.322	-0.605
TYR436	SER224	-3.247	0.845	0.000	0.031	-3.384	-0.738
ASN437	ARG162	-7.338	-12.631	0.000	0.020	-1.327	6.601
TYR440	ASP182	-3.576	-1.333	0.000	0.000	-0.578	-1.665
PRO470	SER199	-3.959	-3.385	0.000	-0.014	-0.622	0.062
	ASP202	-13.641	-11.968	0.000	-0.015	-2.104	0.446
	LEU205	-3.131	-2.718	0.000	0.001	-0.247	-0.168
ALA471	GLY198	-4.446	-4.243	0.000	-0.001	-0.410	0.208
TYR475	SER197	-8.019	-8.226	0.000	1.337	-1.084	-0.046
ASN479	ASP182	-6.089	-2.349	0.000	0.194	-1.679	-2.255
	ALA183	-4.191	-3.523	0.000	-0.028	-0.504	-0.137
ASP480	ARG162	-66.925	-84.021	0.000	-0.951	-1.059	19.106
	SER163	-6.878	-7.213	0.000	0.192	-1.156	1.299
	ASN164	-17.253	-16.828	0.000	0.238	-0.022	-0.641
	ARG223	-36.246	-37.022	0.000	-0.001	-0.521	1.298
TYR481	ARG223	-10.363	-10.219	0.000	0.023	-0.560	0.393
PHE483	ARG223	-11.947	-13.832	0.000	0.011	-0.507	2.382
TYR484	TYR102	-7.166	-0.093	0.000	0.482	-7.717	0.163
THR486	TYR053	-3.473	-2.896	0.000	-0.007	-1.009	0.439
	TYR102	-3.083	-3.027	0.000	0.279	-0.112	-0.222
THR487	SER052	-7.899	-8.074	0.000	0.300	-0.467	0.342
	ASN057	-5.494	-4.710	0.000	-0.003	-0.229	-0.552
	ASP099	-12.832	-12.345	0.000	0.158	-0.690	0.045
GLY488	TYR032	-7.361	-7.584	0.000	-0.111	-0.517	0.850
ILE489	TYR053	-3.313	-1.307	0.000	0.029	-1.301	-0.734
TYR491	ASP099	-8.616	-8.602	0.000	-0.026	-0.875	0.887
	ARG100	-3.640	-0.456	0.000	-0.124	-2.393	-0.666
	ASP105	-4.980	-5.443	0.000	0.000	-0.239	0.701

All energies are in kcal/mol.

The calculation has FMO-DFTB3/D/PCM level

Table S15 The PIEDA Analysis of RBD-SARS-CoV-1/m395 complex (PDB ID: 2DD8)

RBD-SARS-CoV-1	m395	PIE	ΔE^{es}	ΔE^{ex}	ΔE^{ct+mix}	ΔE^{di}	ΔG^{sol}
PHE360	$_{LC}$SER030	-4.866	-3.813	0.000	-0.002	-0.498	-0.552
THR363	$_{LC}$LYS031	-3.640	-8.781	0.000	0.000	-0.405	5.547
LYS365	$_{LC}$ASP092	-31.884	-60.661	0.000	0.000	-0.235	29.012
ASP392	$_{HC}$ASN058	-14.629	-9.496	0.000	0.121	-1.399	-3.855
	$_{LC}$TRP091	-9.780	-9.830	0.000	-0.087	-1.765	1.903
	$_{LC}$SER093	-3.297	-3.908	0.000	0.000	-0.365	0.977
ARG395	$_{LC}$SER093	-4.674	-3.342	0.000	0.000	-0.976	-0.356
	$_{LC}$SER094	-9.769	-6.396	0.000	-0.101	-2.926	-0.346
	$_{LC}$SER095	-3.433	-7.692	0.000	0.002	-1.174	5.431
	$_{LC}$ASP095A	-72.351	-96.594	0.000	-1.514	0.525	25.231
ARG426	$_{HC}$MET098	-6.171	-9.996	0.000	0.000	-0.921	4.747
PHE483	$_{HC}$LEU054	-3.478	-2.609	0.000	0.137	-1.096	0.090
THR487	$_{HC}$TYR032	-8.127	-7.179	0.000	0.010	-1.955	0.997
GLY488	$_{HC}$VAL097	-7.446	-6.571	0.000	-0.027	-1.090	0.243
ILE489	$_{HC}$GLY100	-4.104	-3.867	0.000	0.259	-0.672	0.176
	$_{LC}$TRP091	-4.024	0.303	0.000	0.502	-4.566	-0.262
	$_{LC}$TYR096	-3.331	-1.542	0.000	-0.043	-1.697	-0.049
TYR491	$_{HC}$ALA057	-3.796	-2.688	0.000	-0.418	-0.670	-0.021
	$_{HC}$ASN058	-3.486	-3.188	0.000	1.308	-1.154	-0.452
GLN492	$_{HC}$MET098	-6.430	-6.389	0.000	0.332	0.233	-0.605

All energies are in kcal/mol.

The calculation has FMO-DFTB3/D/PCM level

The subscripts HC and LC indicate the heavy and light chain, respectively

Table S16 The PIEDA Analysis of RBD-SARS-CoV-1/S230 complex A (PDB ID: 6NB6)

RBD-SARS-CoV-1	S230	PIE	ΔE^{es}	ΔE^{ex}	ΔE^{ct+mix}	ΔE^{di}	ΔG^{sol}
^{A}SER432	$_{HC}$ASP101	-5.440	-11.818	0.000	-0.008	-0.714	7.100
^{A}THR486	$_{HC}$ASP101	-5.967	-6.414	0.000	-0.023	-0.583	1.053
^{A}THR487	$_{HC}$ARG104	-9.234	-11.838	0.000	0.021	-0.397	2.979
^{C}VAL404	$_{HC}$TYR106	-5.377	-0.994	0.000	0.121	-4.808	0.305
^{C}ASP407	$_{HC}$ARG104	-33.503	-51.727	0.000	0.000	-0.268	18.493
	$_{HC}$TYR106	-19.127	-18.970	0.000	-1.332	-0.318	1.492
	$_{HC}$HIS109	-3.516	-0.974	0.000	0.000	-0.327	-2.215
^{C}TYR408	$_{HC}$PRO108	-7.369	-7.509	0.000	0.564	-0.342	-0.082
^{C}TYR442	$_{HC}$TYR106	-3.002	-1.476	0.000	0.045	-2.475	0.904
	$_{HC}$PHE107	-4.305	-2.677	0.000	0.009	-1.647	0.010
^{C}LEU443	$_{HC}$PHE107	-3.204	0.193	0.000	-0.026	-3.310	-0.061
^{C}GLY446	$_{LC}$ASP033	-9.701	-9.638	0.000	0.000	-0.346	0.283
^{C}LYS447	$_{LC}$SER032	-3.372	-3.048	0.000	0.224	-0.814	0.265
	$_{LC}$ASP033	-43.634	-69.113	0.000	-0.417	-3.487	29.383
	$_{LC}$GLY034	-14.280	-19.959	0.000	-0.001	-0.950	6.630
^{C}ASP463	$_{LC}$HIS098	-4.811	-0.783	0.000	0.000	-0.234	-3.794
^{C}LYS465	$_{HC}$ASP062	-29.502	-64.610	0.000	-0.001	-0.573	35.681
^{C}CYS467	$_{HC}$PHE059	-4.444	-3.465	0.000	0.058	-1.130	0.093
^{C}LEU472	$_{HC}$TYR060	-3.166	1.029	0.000	0.015	-3.509	-0.700
	$_{HC}$LYS065	-3.084	4.397	0.000	-0.089	-2.485	-4.907
^{C}ASN473	$_{HC}$PHE059	-7.785	-5.582	0.000	-0.037	-2.783	0.617
	$_{HC}$TYR060	-4.975	-3.804	0.000	-0.224	-1.103	0.156
^{C}TYR475	$_{HC}$ASN057	-4.971	-1.337	0.000	-0.109	-3.829	0.304
	$_{HC}$LYS058	-6.041	-2.537	0.000	0.196	-1.185	-2.515

All energies are in kcal/mol.

The calculation has FMO-DFTB3/D/PCM level

The subscripts HC and LC indicate the heavy and light chain, respectively

The superscripts A and C indicate the chain arrangement

Table S17 The PIEDA Analysis of RBD-SARS-CoV-1/S230 complex B (PDB ID: 6NB6)

RBD-SARS-CoV-1	S230	PIE	ΔE^{es}	ΔE^{ex}	ΔE^{ct+mix}	ΔE^{di}	ΔG^{sol}
^{B}ASP407	$_{HC}$ARG104	-25.232	-43.680	0.000	0.000	-0.228	18.676
	$_{HC}$TYR106	-21.025	-20.670	0.000	-1.722	-0.677	2.043
^{B}TYR408	$_{HC}$TYR106	-3.496	-1.233	0.000	0.118	-2.695	0.314
^{B}TYR442	$_{HC}$TYR106	-5.893	-3.879	0.000	-0.123	-2.636	0.745
^{B}GLY446	$_{LC}$ASP033	-3.461	-4.338	0.000	0.000	-0.175	1.052
^{B}LYS447	$_{LC}$SER032	-3.058	-1.217	0.000	0.035	-0.731	-1.144
	$_{LC}$ASP033	-23.107	-70.282	0.000	0.029	-1.823	48.969
^{B}PRO462	$_{LC}$TRP099	-4.230	-0.412	0.000	0.193	-4.021	0.011
^{B}ASP463	$_{LC}$HIS098	-20.442	-22.308	0.000	0.334	-0.565	2.097
	$_{LC}$TRP099	-5.222	-5.121	0.000	0.092	-2.980	2.786
^{B}LYS465	$_{HC}$ASP062	-19.116	-58.960	0.000	0.000	-0.534	40.378
^{B}CYS467	$_{HC}$PHE059	-4.752	-3.673	0.000	0.023	-1.326	0.224
^{B}LEU472	$_{HC}$TYR060	-3.319	1.076	0.000	0.113	-3.975	-0.534
^{B}ASN473	$_{HC}$ALA061	-3.896	-3.753	0.000	0.000	-0.372	0.229
^{B}TYR475	$_{HC}$ASN057	-4.740	-0.995	0.000	0.114	-4.106	0.247

All energies are in kcal/mol.

The calculation has FMO-DFTB3/D/PCM level

The subscripts HC and LC indicate the heavy and light chain, respectively

The superscript B indicates the chain arrangement

Table S18 The PIEDA Analysis of RBD-SARS-CoV-1/S230 complex (PDB ID: 6NB7)

RBD-SARS-CoV-1	S230	PIE	ΔE^{es}	ΔE^{ex}	ΔE^{ct+mix}	ΔE^{di}	ΔG^{sol}
ASP407	$_{HC}$ARG104	-33.182	-50.303	0.000	0.000	-0.320	17.442
GLY446	$_{LC}$ASP033	-4.009	-3.946	0.000	0.000	-0.170	0.106
LYS447	$_{HC}$HIS109	-5.091	-2.637	0.000	0.083	-1.583	-0.955
	$_{LC}$ASP033	-50.387	-84.546	0.000	-0.825	-2.427	37.411
PRO462	$_{LC}$TRP099	-3.571	-0.283	0.000	0.585	-3.653	-0.221
LYS465	$_{HC}$ASP062	-27.929	-72.697	0.000	-0.002	-0.479	45.249
ASN473	$_{HC}$LYS058	-3.002	0.635	0.000	0.000	-0.414	-3.222
	$_{HC}$PHE059	-6.823	-5.605	0.000	-0.123	-1.504	0.409
TYR475	$_{HC}$ASN057	-4.438	-0.670	0.000	0.026	-3.437	-0.358
	$_{HC}$LYS058	-4.394	-6.564	0.000	0.435	-1.063	2.798

All energies are in kcal/mol.

The calculation has FMO-DFTB3/D/PCM level

The subscripts HC and LC indicate the heavy and light chain, respectively

Table S19 The PIEDA Analysis of RBD-SARS-CoV-1/F26G19 complex (PDB ID: 3BGF)

RBD-SARS-CoV-1	F26G19	PIE	ΔE^{es}	ΔE^{ex}	ΔE^{ct+mix}	ΔE^{di}	ΔG^{sol}
PHE360	$_{HC}$THR030	-3.330	-3.105	0.000	0.175	-0.431	0.031
	$_{HC}$GLY054	-3.464	-3.436	0.000	-0.002	-0.376	0.351
SER362	$_{HC}$TYR032	-3.154	-2.985	0.000	0.000	-0.218	0.048
THR363	$_{HC}$THR031	-5.124	-4.606	0.000	-0.074	-0.736	0.291
ASP392	$_{HC}$ILE101	-11.839	-9.745	0.000	-0.031	-2.860	0.797
	$_{HC}$PRO102	-7.170	-6.892	0.000	-0.345	-0.991	1.058
	$_{HC}$GLN103	-14.368	-12.507	0.000	-0.800	-0.944	-0.117
ASP393	$_{HC}$ILE101	-3.943	-4.324	0.000	0.008	-0.422	0.795
ASN424	$_{HC}$TYR052	-3.825	0.330	0.000	0.025	-3.438	-0.742
ARG426	$_{HC}$ASP057	-75.342	-92.492	0.000	-1.159	-0.379	18.689
	$_{HC}$THR059	-8.507	-5.499	0.000	-0.092	-1.888	-1.028
	$_{HC}$TYR060	-10.667	-7.357	0.000	-0.001	-0.258	-3.050
ASN427	$_{HC}$ASP057	-11.098	-15.688	0.000	-0.096	-2.150	6.837
THR486	$_{LC}$PRO095	-4.049	-4.037	0.000	-0.012	-0.854	0.854
THR487	$_{LC}$SER093	-5.091	-3.646	0.000	0.042	-1.736	0.249
	$_{LC}$TYR094	-10.784	-13.838	0.000	3.817	-1.224	0.461
GLY488	$_{LC}$SER093	-6.345	-5.378	0.000	-0.228	-0.998	0.258
	$_{LC}$TYR094	-4.459	-2.216	0.000	-0.080	-2.209	0.047
	$_{HC}$GLU099	-15.107	-14.379	0.000	0.010	-0.445	-0.294
ILE489	$_{HC}$GLU099	-21.464	-19.218	0.000	-0.582	-1.841	0.178
	$_{HC}$ILE101	-5.256	-1.341	0.000	0.079	-3.901	-0.093
TYR491	$_{LC}$TYR032	-4.075	-1.622	0.000	-0.078	-1.921	-0.454
	$_{HC}$GLN103	-9.568	-7.550	0.000	0.014	-2.007	-0.026
GLN492	$_{HC}$GLU099	-8.683	-8.163	0.000	0.000	-0.211	-0.309

All energies are in kcal/mol.

The calculation has FMO-DFTB3/D/PCM level

The subscripts HC and LC indicate the heavy and light chain, respectively

Table S20 The PIEDA Analysis of hACE2/HCoV-NL63 complex (PDB ID: 3KBH)

hACE2	HCoV-NL63	PIE	ΔE^{es}	ΔE^{ex}	ΔE^{ct+mix}	ΔE^{di}	ΔG^{sol}
ASP030	SER496	-6.666	-10.589	0.000	-0.043	-1.587	5.553
LYS031	SER496	-5.353	0.111	0.000	0.282	-0.909	-4.836
ASN033	CYS497	-3.015	-2.998	0.000	0.213	-0.598	0.368
HIS034	GLY495	-6.915	-4.371	0.000	-0.004	-1.518	-1.022
	SER496	-6.370	-2.636	0.000	-0.128	-3.074	-0.532
	CYS497	-7.118	-6.356	0.000	0.003	-1.104	0.340
	HIS503	-5.280	-4.430	0.000	-0.114	-1.120	0.385
GLU037	GLY494	-8.403	-8.234	0.000	0.000	-0.172	0.002
	CYS497	-7.517	-6.299	0.000	-0.008	-0.784	-0.426
	TYR498	-26.534	-25.346	0.000	-1.190	-0.939	0.940
ASP038	GLY494	-4.010	-2.694	0.000	0.000	-0.081	-1.234
MET323	HIS586	-7.212	-7.792	0.000	0.917	-0.161	-0.175
PHE327	PRO536	-3.610	-3.454	0.000	0.372	-1.029	0.501
LYS353	GLY495	-9.948	-11.672	0.000	0.000	-0.347	2.070
	VAL499	-5.048	-6.053	0.000	0.000	-0.303	1.307
GLY354	TYR498	-4.933	-1.722	0.000	0.078	-3.047	-0.242
	SER535	-8.610	-7.992	0.000	-0.314	0.205	-0.509
	GLY537	-4.935	-3.806	0.000	-0.131	-0.679	-0.319
ASP355	SER535	-9.016	-4.846	0.000	-0.132	-0.870	-3.167
	PRO536	-10.167	-9.113	0.000	-0.089	-1.963	0.998
	GLY537	-5.277	-3.292	0.000	-0.013	-1.348	-0.625
	SER540	-5.141	-3.831	0.000	0.000	-0.101	-1.209
	TRP585	-5.352	-4.229	0.000	-0.024	-0.811	-0.288
GLN388	CYS497	-3.659	-3.020	0.000	-0.363	-1.019	0.744
ARG393	VAL499	-8.181	-9.558	0.000	-0.001	-0.517	1.894

All energies are in kcal/mol.

The calculation has FMO-DFTB3/D/PCM level

Table S21 The PIEDA Analysis of hACE2/RBD-SARS-CoV-2 complex (PDB ID: 6M17)

hACE2	RBD-SARS-CoV-2	PIE	ΔE^{es}	ΔE^{ex}	ΔE^{ct+mix}	ΔE^{di}	ΔG^{sol}
GLU023	ALA475	-3.343	-2.872	0.000	0.000	-0.109	-0.362
GLN024	ASN487	-5.459	-5.121	0.000	0.184	-1.004	0.482
PHE028	TYR489	-4.998	-3.161	0.000	0.060	-1.987	0.091
ASP030	LYS417	-15.043	-50.758	0.000	0.000	-0.356	36.071
LYS031	PRO491	-8.249	-11.669	0.000	0.000	-0.235	3.655
	GLN493	-11.211	-16.219	0.000	-0.103	-1.345	6.456
GLU035	GLN493	-14.519	-17.910	0.000	-0.403	-0.342	4.136
GLU037	TYR505	-9.499	-9.379	0.000	-0.105	-0.730	0.715
ASP038	TYR449	-15.242	-12.055	0.000	-1.348	-0.189	-1.650
	GLY496	-12.973	-8.876	0.000	-0.142	-0.467	-3.488
	GLN498	-4.177	-7.991	0.000	0.000	-0.284	4.097
LYS353	PHE497	-14.706	-15.873	0.000	-0.080	-0.660	1.907
GLY354	ASN501	-3.398	-2.827	0.000	-0.002	-0.587	0.017
	GLY502	-4.191	-2.872	0.000	-0.254	-0.487	-0.578
	TYR505	-5.676	-3.658	0.000	0.063	-2.272	0.191
ASP355	THR500	-14.532	-11.818	0.000	-0.025	-0.840	-1.849
	GLY502	-4.665	-4.106	0.000	-0.024	-1.537	1.001
ARG357	ASN501	-10.354	-11.753	0.000	0.000	-0.323	1.722

All energies are in kcal/mol.

The calculation has FMO-DFTB3/D/PCM level

Table S22 The PIEDA Analysis of hACE2/RBD-SARS-CoV-2 complex (PDB ID: 6VW1)

hACE2	RBD-SARS-CoV-2	PIE	ΔE^{es}	ΔE^{ex}	ΔE^{ct+mix}	ΔE^{di}	ΔG^{sol}
SER019	GLY476	-7.213	-6.440	0.000	-0.492	-0.384	0.104
	SER477	-3.790	6.282	0.000	-0.002	-0.942	-9.128
GLN024	ASN487	-5.836	-4.910	0.000	-0.015	-1.666	0.755
ALA025	TYR489	-3.196	-2.932	0.000	-0.008	-0.342	0.085
PHE028	TYR489	-3.069	0.194	0.000	0.057	-3.274	-0.046
LYS031	GLU484	-30.978	-61.223	0.000	0.000	-0.368	30.613
	PRO491	-11.412	-14.523	0.000	-0.002	-0.403	3.516
	GLN493	-19.903	-23.422	0.000	-0.306	-1.011	4.837
GLU035	GLN493	-8.558	-9.261	0.000	-0.930	-1.386	3.019
GLU037	LYS403	-30.142	-45.179	0.000	0.000	-0.144	15.181
	TYR505	-14.640	-14.176	0.000	-1.044	-1.103	1.683
ASP038	TYR449	-21.966	-17.885	0.000	-1.200	0.131	-3.012
	GLY496	-7.046	-10.147	0.000	-0.001	-0.356	3.457
TYR041	GLN498	-3.284	-1.904	0.000	0.030	-1.357	-0.053
	THR500	-3.171	-2.561	0.000	-0.511	-0.204	0.105
	ASN501	-5.265	-3.295	0.000	0.164	-2.335	0.199
GLN042	GLN498	-9.941	-10.508	0.000	0.095	-0.412	0.885
TYR083	PHE486	-3.963	-1.120	0.000	0.117	-3.563	0.603
	ASN487	-6.634	-5.628	0.000	-0.342	-0.772	0.108
ASN090	ARG408	-5.825	0.466	0.000	-0.014	-1.259	-5.018
	THR415	-3.069	-0.467	0.000	0.056	-2.488	-0.170
GLU329	ARG439	-40.043	-81.414	0.000	-0.547	-0.702	42.619
ASN330	ASN501	-4.613	-3.331	0.000	0.069	-0.553	-0.798
LYS353	PHE497	-21.303	-23.152	0.000	-0.397	-0.604	2.850
	GLN498	-8.981	-7.317	0.000	0.000	-0.467	-1.196
	GLY502	-3.590	-2.937	0.000	0.000	-0.371	-0.282
GLY354	ASN501	-6.788	-5.462	0.000	-0.007	-0.835	-0.484
	GLY502	-4.785	-3.688	0.000	-0.093	-0.273	-0.731
	TYR505	-5.875	-3.356	0.000	0.074	-2.563	-0.031
ASP355	THR500	-10.299	-9.936	0.000	-0.005	-0.682	0.325
	GLY502	-4.232	-3.888	0.000	-0.036	-1.533	1.225
ARG357	ASN501	-4.471	-5.545	0.000	0.000	-0.363	1.437

All energies are in kcal/mol.

The calculation has FMO-DFTB3/D/PCM level

Table S23 The PIEDA Analysis of hACE2/RBD-SARS-CoV-2 complex (PDB ID: 6LGZ)

hACE2	RBD-SARS-CoV-2	PIE	ΔE^{es}	ΔE^{ex}	ΔE^{ct+mix}	ΔE^{di}	ΔG^{sol}
SER019	GLY476	-6.565	-8.430	0.000	-0.527	-0.005	2.397
	SER477	-4.382	0.446	0.000	0.005	-0.573	-4.260
GLN024	SER477	-3.511	-1.907	0.000	0.003	-0.684	-0.922
	ASN487	-6.062	-4.680	0.000	-0.160	-1.759	0.537
PHE028	TYR489	-3.460	-0.622	0.000	-0.019	-2.804	-0.014
ASP030	LYS417	-84.907	-107.494	0.000	-2.371	-0.067	25.025
	LEU455	-6.806	-6.478	0.000	-0.062	-1.046	0.780
LYS031	GLU484	-32.480	-61.728	0.000	0.000	-0.356	29.604
	PRO491	-9.025	-12.663	0.000	0.000	-0.315	3.953
	GLN493	-20.912	-24.655	0.000	-0.493	-0.937	5.173
GLU035	GLN493	-9.506	-9.428	0.000	-0.911	-1.408	2.242
GLU037	ARG403	-32.743	-44.173	0.000	0.000	-0.207	11.638
	TYR505	-16.497	-16.112	0.000	-1.191	-1.021	1.828
ASP038	GLY447	-5.061	-2.819	0.000	0.000	-0.092	-2.150
	TYR449	-21.774	-19.123	0.000	-1.711	0.561	-1.501
	GLN493	-4.491	-5.008	0.000	0.000	-0.169	0.685
	GLY496	-12.419	-14.669	0.000	-0.172	-0.566	2.988
	GLN498	-4.043	-4.269	0.000	-0.001	-0.286	0.513
TYR041	GLN498	-3.601	-0.712	0.000	0.001	-2.896	0.007
	ASN501	-4.526	-2.337	0.000	0.057	-2.389	0.142
GLN042	GLN498	-3.148	-1.289	0.000	0.093	-0.877	-1.075
TYR083	PHE486	-3.961	-1.488	0.000	0.157	-3.151	0.521
	ASN487	-7.246	-6.669	0.000	0.052	-0.742	0.113
ASN330	ASN501	-3.617	-2.931	0.000	0.275	-0.097	-0.865
LYS353	PHE497	-20.362	-20.042	0.000	0.004	-1.259	0.935
GLY354	ASN501	-5.622	-4.338	0.000	-0.010	-0.803	-0.471
	GLY502	-4.650	-3.744	0.000	0.065	-0.285	-0.686
	TYR505	-6.810	-4.146	0.000	0.078	-2.740	-0.002
ASP355	THR500	-10.068	-9.434	0.000	-0.001	-0.528	-0.105
	GLY502	-3.302	-2.924	0.000	-0.053	-1.580	1.255
ARG357	ASN501	-4.400	-5.330	0.000	-0.001	-0.436	1.368

All energies are in kcal/mol.

The calculation has FMO-DFTB3/D/PCM level

Table S24 The PIEDA Analysis of hACE2/RBD-SARS-CoV-2 complex (PDB ID: 6M0J)

hACE2	RBD-SARS-CoV-2	PIE	ΔE^{es}	ΔE^{ex}	ΔE^{ct+mix}	ΔE^{di}	ΔG^{sol}
SER019	GLY476	-5.072	-7.533	0.000	-0.001	-0.363	2.824
GLN024	ASN487	-6.567	-5.396	0.000	-0.137	-1.627	0.592
PHE028	TYR489	-3.327	-0.457	0.000	0.001	-2.811	-0.059
ASP030	LYS417	-82.921	-108.320	0.000	-2.173	-0.066	27.638
	LEU455	-7.375	-6.979	0.000	-0.148	-1.024	0.776
LYS031	GLU484	-42.876	-72.495	0.000	-0.003	-0.608	30.230
	PRO491	-9.975	-11.183	0.000	0.000	-0.206	1.414
	GLN493	-8.409	-13.422	0.000	-0.030	-1.413	6.456
GLU037	TYR505	-16.703	-16.088	0.000	-1.233	-1.030	1.647
ASP038	TYR449	-20.325	-18.742	0.000	-1.977	0.714	-0.319
	GLY496	-11.811	-14.151	0.000	-0.076	-0.544	2.960
	GLN498	-4.893	-6.252	0.000	-0.064	-0.452	1.875
TYR041	GLN498	-3.013	-0.252	0.000	0.096	-2.846	-0.011
	ASN501	-4.644	-2.450	0.000	0.083	-2.474	0.197
TYR083	PHE486	-4.053	-1.297	0.000	0.205	-3.446	0.485
	ASN487	-6.590	-5.798	0.000	-0.169	-0.750	0.127
ASN330	ASN501	-4.612	-3.348	0.000	0.079	-0.630	-0.712
LYS353	PHE497	-23.481	-26.190	0.000	0.462	-0.212	2.459
	GLN498	-10.409	-8.191	0.000	-0.155	-1.185	-0.879
	GLY502	-3.651	-2.986	0.000	0.000	-0.356	-0.308
GLY354	ASN501	-6.356	-5.012	0.000	-0.006	-0.845	-0.493
	GLY502	-3.818	-2.901	0.000	0.031	-0.185	-0.762
	TYR505	-6.401	-3.675	0.000	0.070	-2.643	-0.154
ASP355	THR500	-11.049	-10.097	0.000	-0.004	-0.696	-0.251
	GLY502	-4.265	-3.899	0.000	0.004	-1.521	1.151
ARG357	ASN501	-4.311	-5.356	0.000	0.000	-0.390	1.436

All energies are in kcal/mol.

The calculation has FMO-DFTB3/D/PCM level

Table S25 The PIEDA Analysis of RBD-SARS-CoV-2/B38 complex (PDB ID: 7BZ5)

RBD-SARS-CoV-2	B38	PIE	ΔE^{es}	ΔE^{ex}	ΔE^{ct+mix}	ΔE^{di}	ΔG^{sol}
ARG403	$_{LC}$GLN090	-3.994	-3.567	0.000	0.000	-0.105	-0.322
	$_{LC}$SER093	-9.831	-9.063	0.000	0.064	-0.562	-0.269
ASP405	$_{LC}$TYR094	-13.309	-9.642	0.000	0.231	-3.063	-0.836
GLU406	$_{LC}$TYR094	-7.647	-3.735	0.000	-0.019	-1.907	-1.987
THR415	$_{HC}$TYR058	-3.822	-2.890	0.000	-0.040	-0.530	-0.363
LYS417	$_{HC}$TYR052	-6.442	-1.136	0.000	-0.520	-3.401	-1.385
	$_{HC}$GLU098	-37.437	-58.252	0.000	0.000	-0.204	21.018
	$_{LC}$ASN092	-16.891	-8.025	0.000	-0.905	-1.459	-6.501
ASP420	$_{HC}$TYR052	-8.738	-8.215	0.000	-0.040	-0.838	0.354
	$_{HC}$GLY054	-5.494	-5.378	0.000	0.000	-0.139	0.022
	$_{HC}$SER056	-28.257	-28.383	0.000	-0.630	0.826	-0.070
TYR421	$_{HC}$TYR052	-3.760	-1.780	0.000	-0.099	-1.899	0.018
PHE456	$_{HC}$TYR033	-9.143	-8.310	0.000	0.553	-1.667	0.281
	$_{HC}$TYR100	-3.903	-1.044	0.000	-0.048	-2.760	-0.052
LYS458	$_{HC}$SER030	-6.177	-3.871	0.000	0.093	-0.789	-1.611
	$_{HC}$SER031	-11.578	-8.480	0.000	-1.453	-1.987	0.342
	$_{HC}$ASN032	-11.910	-12.445	0.000	-0.041	-0.560	1.137
	$_{HC}$GLY054	-10.604	-10.650	0.000	0.000	-0.648	0.694
	$_{HC}$ASN073	-9.619	0.529	0.000	0.000	-0.213	-9.935
SER459	$_{HC}$SER053	-3.477	-2.825	0.000	0.002	-0.646	-0.008
ASN460	$_{HC}$GLY054	-5.278	-4.196	0.000	-0.201	-0.906	0.024
TYR473	$_{HC}$ASN032	-5.654	-6.773	0.000	0.922	0.425	-0.228
ALA475	$_{HC}$ASN032	-3.908	-2.559	0.000	-0.010	-1.035	-0.305
GLY476	$_{HC}$ILE028	-5.941	-4.103	0.000	-0.528	-1.336	0.025
	$_{HC}$SER031	-3.766	-3.025	0.000	0.002	-0.753	0.012
SER477	$_{HC}$ILE028	-4.805	-2.401	0.000	0.058	-2.045	-0.418
GLU484	$_{HC}$TYR100	-4.784	-3.983	0.000	0.000	-0.200	-0.601
PHE486	$_{HC}$ASP103	-4.134	-3.769	0.000	-0.008	-0.762	0.405
ASN487	$_{HC}$PHE027	-4.457	-4.084	0.000	0.968	-1.135	-0.205
	$_{HC}$ARG097	-10.312	-12.199	0.000	0.061	-0.151	1.978
TYR489	$_{HC}$ASP103	-3.959	-3.450	0.000	-0.001	-0.340	-0.168
GLN493	$_{HC}$TYR100	-3.393	-1.745	0.000	0.011	-1.864	0.205
GLY496	$_{LC}$TYR032	-6.697	-6.539	0.000	-0.105	0.059	-0.112
PHE497	$_{LC}$SER030	-4.606	-4.206	0.000	-0.174	-0.272	0.046
ASN501	$_{LC}$GLY028	-4.119	-3.928	0.000	-0.169	-0.781	0.759
	$_{LC}$SER030	-3.186	-2.093	0.000	0.052	-1.246	0.100
GLY502	$_{LC}$ILE029	-9.854	-9.734	0.000	-0.088	0.086	-0.118
VAL503	$_{LC}$GLN027	-3.689	-3.037	0.000	-0.010	-0.654	0.012
TYR505	$_{LC}$ILE029	-6.139	-1.829	0.000	-0.054	-4.301	0.045
	$_{LC}$TYR032	-3.166	0.085	0.000	-0.013	-3.288	0.049
	$_{LC}$GLN090	-8.870	-7.579	0.000	-0.780	-0.477	-0.034

All energies are in kcal/mol.

The calculation has FMO-DFTB3/D/PCM level
